# The Anticancer Activities Phenolic Amides from the Stem of *Lycium barbarum*

**DOI:** 10.1007/s13659-017-0134-x

**Published:** 2017-06-06

**Authors:** Pei-Feng Zhu, Zhi Dai, Bei Wang, Xin Wei, Hao-Fei Yu, Zi-Ru Yan, Xu-Dong Zhao, Ya-Ping Liu, Xiao-Dong Luo

**Affiliations:** 1 State Key Laboratory of Phytochemistry and Plant Resources in West China, Kunming Institute of Botany, Chinese Academy of Sciences, Kunming, 650201 People’s Republic of China; 2 University of Chinese Academy of Sciences, Beijing, 100049 People’s Republic of China; 3 Yunnan Key Laboratory of Natural Medicinal Chemistry, Kunming, 650201 People’s Republic of China; 4 Key Laboratory of Animal Models and Human Disease Mechanisms of Chinese Academy of Sciences/Key Laboratory of Bioactive Peptides of Yunnan Province, Kunming Institute of Zoology, Kunming, 650223 Yunnan China

**Keywords:** *Lycium barbarum*, Phenolic amides, Anticancer activities, Glioma stem cell

## Abstract

**Abstract:**

Four new phenolic amides, 4-O-methylgrossamide (**1**), (*E*)-2-(4,5-dihydroxy-2-{3-[(4-hydroxyphenethyl)amino]-3-oxopropyl}-phenyl)-3-(4-hydroxy-3-methoxyphenyl)-N-(4-hydroxyphenethyl)acryl-amide (**2**), (*Z*)-lyciumamide C (**3**), (*Z*)-thoreliamide B (**4**), together with thirteen known phenolic amides were identified from the stem of *Lycium barbarum.* The structures of the new compounds were determined by spectroscopic methods. All compounds were evaluated for their anti-cancer activities against human glioma stem cell lines.

**Graphical Abstract:**

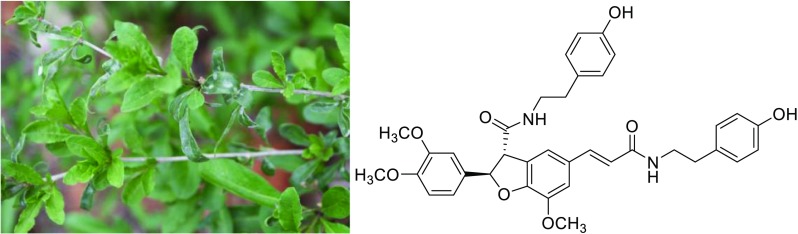

**Electronic supplementary material:**

The online version of this article (doi:10.1007/s13659-017-0134-x) contains supplementary material, which is available to authorized users.

## Introduction

**Fig. 1:**
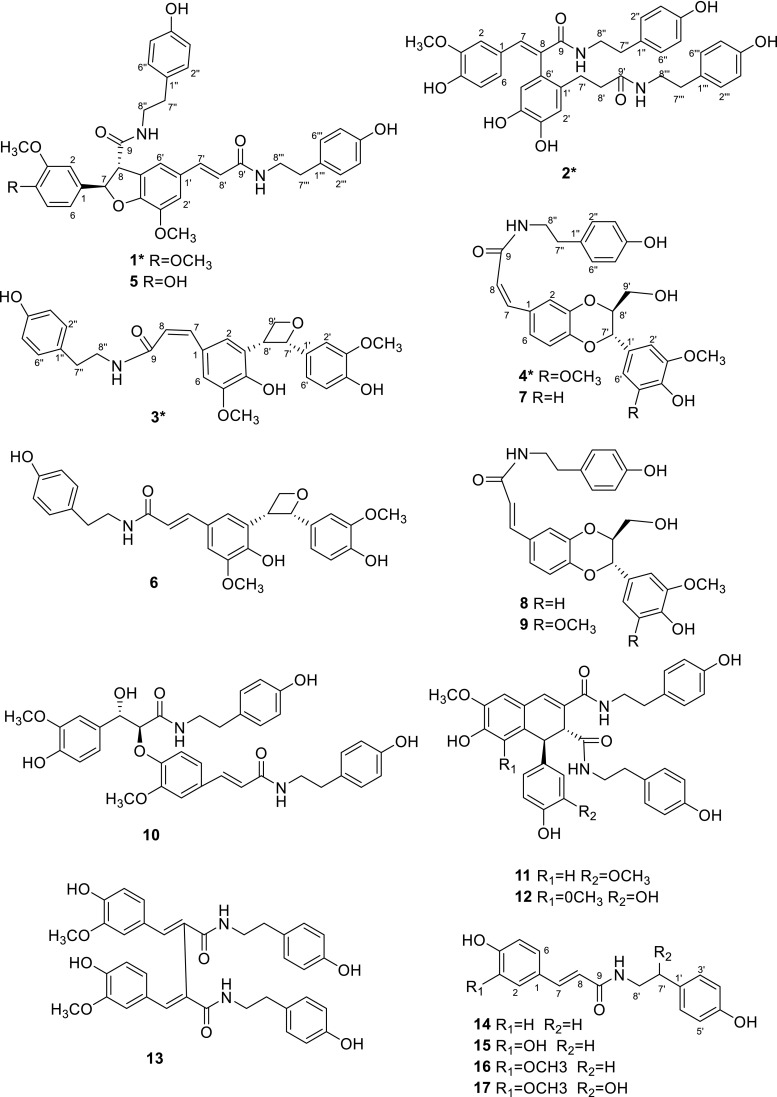
Structures of compounds **1**–**17**

*Lycium barbarum* had a long history of usage as a traditional herbal medicine and functional food in Asian countries [1]. Besides, its fruits known as goji or wolf berries, were beneficial to human health and very important agricultural products [2, 3]. Modern pharmacological studies indicated *L. barbarum* possessed widely health-promoting and medical effects, including antioxidant [4], lipotropic [5], hepatic function protecting effects [5], immunomodulatory properties [6], antiaging [7], anticancer activities [8–11] and so on. Phytochemical studies showed that phenolic amides were not only characteristic compounds but also abundant ones in *L. Barbarum* [2]. Phenolic amides, originating from the condensation of cinnamic acid derivatives and tyramines, octopamines or aliphatic amines [2, 12], had been reported a range of biological activities, like antioxidant [4], antiobesity [13], cytotoxicity [14], anti-inflammatory activity [15] and potent inhibitors of de novo nucleotide biosynthesis [16], and they also seemed to play an important role in plant defense against pathogens [12]. Continuation of our study on the phenolic amides had led to the isolation of four new phenolic amides, 4-O-methylgrossamide (**1**), (*E*)-2-(4,5-dihydroxy-2-{3-[(4-hydroxyphenethyl)amino]-3-oxopropyl}-phenyl)-3-(4-hydroxy-3-methoxyphenyl)-N-(4-hydroxyphenethyl)acryl-amide (**2**), (*Z*)-lyciumamide C (**3**), (*Z*)-thoreliamide B (**4**), together with thirteen known phenolic amides (Fig. 1) from *L. barbarum.* The known compounds were identified as grossamide (**5**) [17], lyciumamide C (**6**) [4], (*Z*)-3-{(2,3-*trans*)-2-(4-hydroxy-3-methoxy-phenyl)-3-hydroxymethyl-2,3-dihydrobenzo[1, 4]-dioxin-6-yl}-N-(4-hydroxyphenethyl)acrylamide (**7**), [18] (*E*)-3-{(2,3-*trans*)-2-(4-hydroxy-3-methoxyphenyl)-3-hydroxy-methyl-2,3-dihydrobenzo[1, 4]dioxin-6-yl}-N-(4-hydroxyphenethyl)acryl-amide (**8**) [18], (*E*)-thoreliaide B (**9**) [19], cannabisin E (**10**) [20], cannabisin D (**11**) [21], 1,2-dihydro-6,8-dimethoxy-7-hydroxyl-(3,5-dimethoxy-4-hydroxyphenyl)-N′,N-2-bis[2-(4-hydroxyphenyl)ethyl]-2,3-naphthal-enedicarboxamide (**12**) [22], cannabisin G (**13**) [20], N-*E*-p-coumaroyl tyramine (**14**) [23], N-*E*-caffeoyl tyramine (**15**) [24], N-*E*-feruloyl tyramine (**16**) [25], N-*E*-feruloyl octopamine (**17**) [25], by comparison with the data in the literature values. All of the compounds were evaluated for anti-cancer activity against human glioma stem cell lines, and compounds **1** and **5** exhibited moderate anti-cancer activities. Herein the isolation, structural elucidation and the bioactivity of the phenolic compounds were reported.

## Results and Discussion

Compound **1** was obtained as a white powder. Its molecular formula was determined to be C_37_H_38_N_2_O_8_ on the basis of its ^13^C NMR and HRESIMS (m/z 639.2721 [M+H]^+^, calcd. for C_37_H_39_N_2_O_8_, 639.2701), suggesting 20 degrees of unsaturation. The UV maxima (249, 289 and 320 nm) showed the existence of aromatic rings [19]. The IR spectrum indicated the presence of OH (3387 cm^−1^), aromatic rings (1651, 1605, 1517 cm^−1^), and C–N bond (1265 cm^−1^) groups [14]. In the ^1^H NMR spectrum, four pairs symmetrical aromatic protons signals at *δ*_H_ 7.04 (2H, d, *J* = 8.4 Hz), 6.71 (2H, d, *J* = 8.4 Hz), 7.01 (2H, d, *J* = 8.5 Hz), and 6.69 (2H, *J* = 8.5 Hz) were readily assigned to two AA′BB′ system of **1** [26]. Four pairs of vicinal methylenes protons signals at *δ*_H_ 2.67 (4H, t, *J* = 5.1 Hz), 3.36 (4H, t, *J* = 5.1 Hz), along with four methylenes carbons signals at *δ*_C_ 34.4 (t), 40.9 (t), 34.2 (t), and 40.8 (t) were the characteristic resonances for two –NHCH_2_CH_2_– groups of **1** [19]. In the ^13^C NMR spectrum, two carbonyl carbon resonances at *δ*_C_ 169.4 (s) and 165.2 (s) were readily assigned to two amidic moieties, which indicated by HMBC correlations from *δ*_H_ 5.93 (1H, d, *J* = 8.1 Hz, H-7) and 3.36 (2H, t, *J* = 5.1 Hz, H-8″) to *δ*_C_ 169.4 (s, C-9), from *δ*_H_7.38 (1H, d, *J* = 15.7 Hz, H-7′) and 3.36 (2H, t, *J* = 5.1 Hz, H-8‴) to *δ*_C_ 165.2 (s, C-9′). According to these 1D NMR data (Table 1), compound **1** was readily identified as a phenolic amide with two tyramine moieties [18]. Detailed analysis of 1D NMR spectra of **1** displayed similarities to those of **5** [27], except for a methoxy at C-4 in **1** instead of a hydrogen at C-4 in **5**, which indicated by HMBC correlations from *δ*_H_ 3.76 (3H, s, 4-OCH_3_), 6.85 (1H, dd, *J* = 8.4, 2.0 Hz, H-6) and 6.94 (1H, d, *J* = 2.0 Hz, H-2) to *δ*_C_ 149.0 (s, C-4), from *δ*_H_ 3.78 (3H, s, 3-OCH_3_) and 6.98 (1H, d, *J* = 8.4 Hz, H-5) to *δ*_C_ 148.9 (s, C-3).

**Table 1:** ^1^H (600 MHz) and ^13^C NMR (150 MHz) Data for compounds **1**, **2** in DMSO-*d*_6_ and **4** in CD_3_OD (*δ* in ppm, *J* in Hz)

No.	**1**		**2**		**4**	
	*δ*_H_	*δ*_C_	*δ*_H_	*δ*_C_	*δ*_H_	*δ*_C_
**1**		132.1		126.5		130.1
**2**	6.94 d (2.0)	109.6	6.39 d (2.0)	112.4	7.21 d (2.1)	119.0
**3**		148.9		147.4		144.8
**4**		149.0		146.9		145.6
**5**	6.98 d (8.4)	111.7	6.66 d (8.3)	115.2	6.92 d (8.4)	117.8
**6**	6.85 dd (8.4, 2.0)	118.4	6.73 dd (8.3, 2.0)	124.7	6.98 dd (8.4, 2.1)	124.5
**7**	5.93 d (8.1)	87.5	7.57 s	135.0	6.63 d (12.6)	137.2
**8**	4.23 d (8.1)	56.0		125.6	5.88 d (12.6)	123.3
**9**		169.4		166.8		170.4
**3-OMe**	3.78 s	55.7	3.37 s	54.6		
**4-OMe**	3.76 s	55.5				
**1′**		128.6		130.7		128.6
**2′**	7.16 d (1.6)	111,.7	6.52 s	116.7	6.70 br. s	106.0
**3′**		144.1		144.4		149.5
**4′**		148.7		145.5		137.7
**5′**		128.4	6.80 s	116.6		149.5
**6′**	6.91 s	115.9		131.4	6.70 br. s	106.0
**7′**	7.38 d (15.7)	138.7	2.51 m 2.43 m	27.9	4.88 d (8.1)	78.0
**8′**	6.49 d (15.7)	119.7	2.13 td (9.4, 6.5)	36.0	4.07 ddd (8.1, 4.3, 2.5)	80.3
**9′**		165.2		171.4	3.71 dd (12.5, 2.5) 3.48 dd (12.5, 4.3)	62.2
**3′-OMe**	3.85 s	55.7			3.75 s	56.9
**1″**		129.7		129.6		131.3
**2″**	7.04 d (8.4)	129.6	6.94 d (8.4)	129.5	6.98 d (8.4)	130.8
**3″**	6.71 d (8.4)	115.2	6.68 d (8.4)	115.1	6.67 d (8.4)	116.4
**4″**		155.7		155.7		157.1
**5″**	6.71 d (8.4)	115.2	6.68 d (8.4)	115.1	6.67 d (8.4)	116.4
**6″**	7.04 d (8.4)	129.6	6.94 d (8.4)	129.5	6.98 d (8.4)	130.8
**7″**	2.67 t (5.1)	34.4	2.66 t (7.5)	34.6	2.68 t (7.6)	35.5
**8″**	3.36 t (5.1)	40.9	3.41 t (7.5)	41.5	3.41 m	42.5
**1‴**		129.3		129.6		
**2‴**	7.01 d (8.5)	129.5	6.91 d (8.4)	129.4		
**3‴**	6.69 d (8.5)	115.1	6.65 d (8.4)	115.1		
**4‴**		155.7		155.6		
**5‴**	6.69 d (8.5)	115.1	6.65 d (8.4)	115.1		
**6‴**	7.01 d (8.5)	129.5	6.91 d (8.4)	129.4		
**7‴**	2.67 t (5.1)	34.2	2.58 t (7.4)	34.5		
**8‴**	3.36 t (5.1)	40.8	3.20 t (7.4)	40.7		

The large coupling constants of H-7′ with H-8′ (*J* = 15.7 Hz) suggested the configuration of the double bond at C-7′/C-8′ was *E* [17]. The configuration of H-7 with H-8 was *threo*, which was assigned by the ROESY correlations of H-8 and H-6 (Fig. 3), while there was no REOSY correlation between H-7 and H-8 [28]. The absolute configuration at C-7 and C-8 were 7R, 8S, respectively, which determined by the negative cotton effect at 257 nm (Δε − 0.45) observed in circular dichroic spectrum [28–31]. Detailed analysis of 2D NMR data (HSQC, HMBC, ROESY) established the structure of **1** to be as shown, named 4-O-methylgrossamide.

**Fig. 2:**
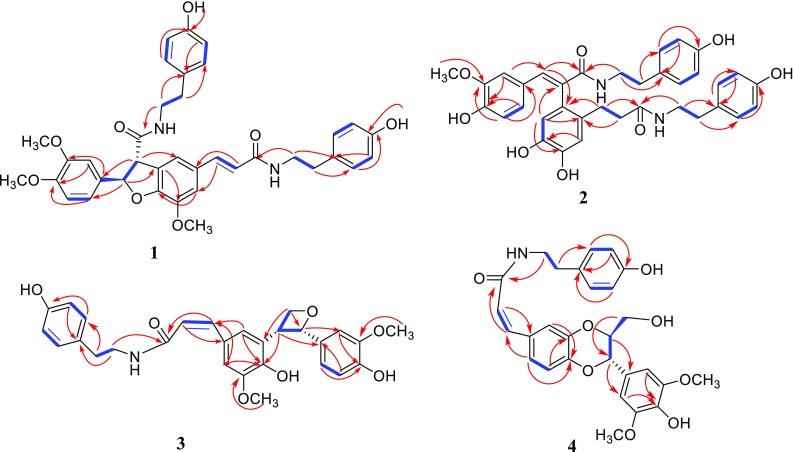
Key ^1^H–^1^H COSY () and HMBC () correlations for compounds **1**–**4**

**Fig. 3:**
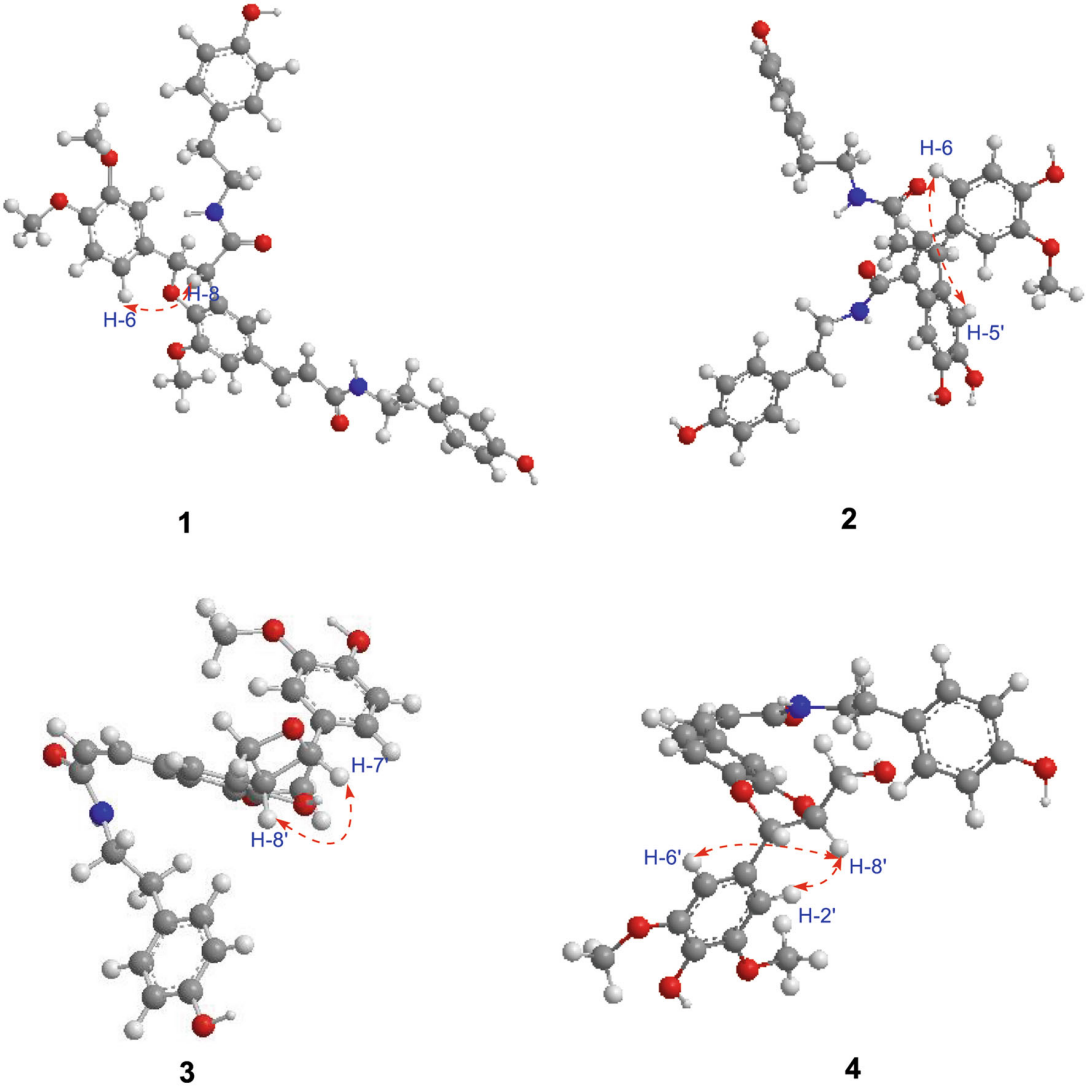
Key ROESY () correlations of compounds **1**–**4**

Compound **2** was obtained as a yellow powder. Its molecular formula C_35_H_36_N_2_O_8_ was established by ^13^C NMR and positive HR–ESI–MS data ([M+Na]^+^ 635.2368, calcd. for C_35_H_36_N_2_O_8_Na, 635.2364). The IR spectrum absorptions showed the existence of OH (3422 cm^−1^), conjugated C=O (1636 cm^−1^) and Ph (1614 and 1514 cm^−1^) groups [19]. In the 1D-NMR spectrum, four pairs symmetrical aromatic protons at *δ*_H_ 6.94 (2H, d, *J* = 8.4 Hz), 6.68 (2H, d, *J* = 8.4 Hz), 6.91 (2H, d, *J* = 8.4 Hz), and 6.65 (2H, d, *J* = 8.4 Hz) suggested the presence of two AA′BB′ systems [26]. Four pairs of vicinal methylenes protons signals at *δ*_H_ 2.66 (2H, t, *J* = 7.5 Hz), 3.41 (2H, t, *J* = 7.5 Hz), 2.58 (2H, t, *J* = 7.4 Hz), and 3.20 (2H, t, *J* = 7.4 Hz), along with two carbonyl carbons at *δ*_C_ 171.4 (s), and 166.8 (s) were the characteristic resonances for two –CONHCH_2_CH_2_– moieties, which were supported by HMBC correlations of *δ*_H_ 7.57 (1H, s, H-7), 3.41 (2H, t, *J* = 7.5 Hz, H-8″) with *δ*_C_ 166.8 (s, C-9), of *δ*_H_ 2.43 (1H, m, H-7′), 3.20 (2H, t, *J* = 7.4 Hz, H-8‴) with *δ*_C_ 171.4 (s, C-9′) [19]. Analysis of the ^1^H, ^13^C NMR data (Table 1) indicated that **2** was similar to those of (*E*)-2-(4,5-dihydroxy-2-{3-[(4-hydroxyphenethyl)amino]-3-oxopropyl}-phenyl)-3-(4-hydroxy-3,5-dimethyoxyphenyl)-N-(4-hydroxyphenethyl)-acrylamide [18], except for the missing of –OCH_3_ at C-5 in **2**, as supported by the highfield shifted at C-5 (from *δ*_C_ 148.7 in the forementioned to *δ*_C_ 115.2 in **2**) and the HMBC correlations from 6.66 (1H, d, *J* = 8.3 Hz, H-5) with *δ*_C_ 126.5 (s, C-1) and 147.4 (s, C-3). The only methoxyl was substituted at C-3, which was supported by the HMBC correlations of *δ*_H_ 3.37 (3H, s), 6.66 (1H, d, *J* = 8.3 Hz, H-5) with *δ*_C_ 147.4 (s, C-3). The HMBC correlations of *δ*_H_ 2.43 (1H, m, H-7′) with *δ*_C_ 171.4 (s, C-9′), 131.4 (s, C-6′), and 116.7 (d, C-2′), of *δ*_H_ 2.13 (2H, td, *J* = 9.4, 6.5 Hz, H-8′) with *δ*_C_ 130.7 (s, C-1′), suggested the –CH_2_CH_2_– fragment was connected between C-1′ and C-9′ (Fig. 2). ROESY correlation of H-6 with H-5′ (Fig. 3) suggested two benzene rings, connected to C-7 and C-8 respectively, were on the same side, further confirmed the double bond of C-7/C-8 was *E* [18]. Hence, Detailed analysis of 2D NMR data established the structure of compound **2** to be as shown, named (*E*)-2-(4,5-dihydroxy-2-{3-[(4-hydroxyphenethyl)amino]-3-oxopropyl}phenyl)-3-(4-hydroxy-3-methoxy-phenyl)-N-(4-hydroxyphenethyl)acrylamide.

The other phenolic amide, **3**, obtained as a white powder, with the same R_*f*_ value on silica plate as that of **6**, showed identical physical data in the HRESIMS and IR spectra, indicating the existence of the same molecular formula and functional groups as in **6** [4]. The 1D-NMR displayed an AA′BB′ system [26] [*δ*_H_ 6.96 (2H, d, *J* = 8.4 Hz), and 6.68 (2H, d, *J* = 8.5 Hz), along with *δ*_C_ 130.8 (d), and 116.4 (d)], five proton signals [(*δ*_H_ 7.22 (1H, d, *J* = 1.7 Hz), 7.05 (1H, s), 6.93 (1H, d, *J* = 2.0 Hz), 6.81 (1H, dd, *J* = 8.1, 2.0 Hz) and 6.75 (1H, d, *J* = 8.1 Hz)] suggested the existence of two aromatic rings [18]. The ^13^C NMR also showed an amidic carbon [*δ*_C_ 170.3 (s, C-9) and *δ*_C_ 42.5 (t, C-8″)], which indicated by HMBC correlations from *δ*_H_ 6.66 (1H, d, *J* = 12.6 Hz, H-7) and 3.31 (2H, t, *J* = 7.5 Hz, H-8″) to *δ*_C_ 170.3 (s, C-9) (Fig. 2).

Detailed analysis of 1D, 2D NMR spectral data (Table 2) suggested that the planar structure of **3** was the same as **6**. The visible difference was that the configuration of the double bond at C-7/C-8 in **3** was *Z*, which was suggested by coupling constants (*J* = 12.6 Hz) between H-7 and H-8 [4, 25]. The NOESY correlations of H-7′ and H-8′ suggested the configuration of H-7′/H-8′ was *erythro* (Fig. 3) [4], which also supported by the specific optical rotation of **3** $[[\upalpha]_{\text{D}}^{25}$ − 5.0° (c 0.11, MeOH)] was the same side as that of **6** $[[\upalpha]_{\text{D}}^{22}$ − 7.9° (c 0.34, MeOH)] [4]. Other parts of the structure were identical to those of **6** by detailed analysis of its 2D NMR spectra. Thus, the structure of **3** was established and named *Z*-lyciumamide C.

**Table 2:** ^1^H (500 MHz) and ^13^C NMR (125 MHz) Data for compounds **3** and **6** in DMSO-*d*_6_ and CD_3_OD (*δ* in ppm, *J* in Hz)

No.	3 (in DMSO-*d*_6_)		3 (in CD_3_OD)		6 (in DMSO-*d*_6_)		6 (in CD_3_OD)	
	*δ*_H_	*δ*_C_	*δ*_H_	*δ*_C_	*δ*_H_	*δ*_C_	*δ*_H_	*δ*_C_
**1**		128.7		130.5		128.5		130.8
**2**	7.60 s	114.4	7.22 d (1.7)	115.4	7.10 s	111.9	7.07 d (1.5)	113.2
**3**		142.9		145.2		143.9		145.7
**4**		148.1		150.2		149.0		151.3
**5**		128.7		129.9		130.1		130.2
**6**	7.23 s	119.7	7.05 s	120.2	7.16 s	116.6	7.12 s	116.2
**7**	6.55 d (12.9)	136.7	6.66 d (12.6)	138.5	7.38 d (15.7)	139.1	7.46 d (15.7)	141.9
**8**	5.81 d (12.9)	121.5	5.85 d (12.6)	122.3	6.50 d (15.7)	119.4	6.42 d (15.7)	119.7
**9**		166.2		170.3		165.4		169.0
**5-OMe**	3.73 s	55.6	3.78 s	56.5	3.73 s	55.6	3.80 s	56.4
**1′**		132.2		134.5		132.0		134.1
**2′**	6.90 s	110.3	6.93 d (2.0)	110.6	6.94 s	110.4	6.93 d (2.0)	110.5
**3′**		147.6		149.2		147.7		149.1
**4′**		146.4		147.7		146.6		147.7
**5′**	6.75 s	115.3	6.75 d (8.1)	116.3	6.79 s	115.4	6.77 d (8.2)	116.2
**6′**	6.75 s	118.5	6.81 dd (8.1, 2.0)	119.8	6.79 s	118.8	6.81 dd (8.2,2.0)	118.5
**7′**	5.50 d (6.5)	87.6	5.56 d (6.2)	89.7	5.52 d (6.9)	87.7	5.55 d (6.4)	89.7
**8′**	3.46 d (6.5)	52.8	3.41 d (5.8)	55.2	3.49 d (6.9)	52.7	3.45 dd (8.0,6.7)	54.8
**9′**	3.61 dd (10.6,7.2)	63.1	3.82 d (4.8)	64.9	3.71 dd (10.6,6.0)	62.8	3.83 d (5.2)	64.6
	3.69 dd (10.6,4.8)		3.75 d (6.1)		3.69 dd (10.6,4.8)			
**3′-OMe**	3.76 s	55.6	3.85 s	56.8	3.77 s	55.7	3.87 s	56.7
**1″**		129.5		131.3		129.5		131.2
**2″**	6.98 d (8.5)	129.4	6.96 d (8.4)	130.8	7.04 d (8.6)	129.6	7.04 d (8.4)	130.7
**3″**	6.67 d (8.4)	115.1	6.68 d (8.5)	116.4	6.71 d (8.4)	115.2	6.71 d (8.5)	116.3
**4″**		155.6		157.1		155.7		156.9
**5″**	6.67 d (8.4)	115.1	6.68 d (8.5)	116.4	6.71 d (8.4)	115.2	6.71 d (8.5)	116.3
**6″**	6.98 d (8.5)	129.4	6.96 d (8.4)	130.8	7.04 d (8.6)	129.6	7.04 d (8.4)	130.7
**7″**	2.61 t (7.5)	34.2	2.65 t (7.5)	35.9	2.67 t (7.4)	34.4	2.74 t (7.3)	35.8
**8″**	3.26 t (7.5)	40.6	3.31 t (7.5)	42.5	3.35 t (7.4)	40.8	3.45 t (7.3)	42.5

The aromatic rings of the phenylpropanoid units exhibited various oxygenation patterns, and it had been summarized that the deoxygenation patterns might involve the 3,4-, 2,4-, 2,5- and 2,6-positions [4, 17, 19–21], but seldom the 3,5-positions [18]. Some compounds which had 3,5-positions substituents always been proven to be 3,4- 2,4- or 2,5-positions substituents, owing to the existence of “deceptively simple” protons signals when changed the deuterated solvent or temperature [32]. Interestingly, in our experiment, the “deceptively simple” protons signals that exhibited two broad singlets with approximate integrations of 1:2 for H-2′ and for H-5′, H-6′ were found when using DMSO-*d*_6_, and they would be a set of proton signals with a *m*-coupling constant for H-2′ (d, *J* = 2.0 Hz), an *o*-coupling constant for H-5′ (d, *J* = 8.1 Hz), and *o*-,*m*-coupling constants for H-6′ (dd, *J* = 8.1, 2.0 Hz) in **3**, and with a *m*-coupling constant for H-2′ (d, *J* = 2.0 Hz), an *o*-coupling constant for H-5′ (d, *J* = 8.2 Hz), and *o*-,*m*-coupling constants for H-6′ (dd, *J* = 8.2, 2.0 Hz) in **6** when using CD_3_OD (SI, Figs. S37, S38).

Compound **4** was obtained as faint yellow powder. Its molecular formula C_28_H_29_NO_8_ was established by ^13^C NMR and positive ESIMS m/z 508 [M+H]^+^. The UV maxima (207, 240 and 278 nm) showed the existence of aromatic rings and IR bands (3421, 1648, 1614, 1512, and 1274 cm^−1^) displayed aromatic rings, hydroxyl functions and a C–N bond [18]. Analysis of the ^1^H, ^13^C NMR data (Table 1) revealed that compounds **4** and **9** are structurally similar except for the configuration of the double bone at C-7/C-8. The coupling constants (*J* = 12.6 Hz) between H-7 and H-8 suggested the configuration of the double bone at C-7/C-8 in **4** was *Z* [21]. The ^1^H–^1^H COSY correlations of *δ*_H_ 4.88 (1H, d, *J* = 8.1 Hz, H-7′)/4.07 (1H, ddd, *J* = 8.1, 4.3, 2.5 Hz, H-8′)/3.71 (1H, dd, *J* = 12.5, 2.5 Hz, H-9′)/3.48 (1H, dd, *J* = 12.5, 4.3 Hz, H-9′) revealed the presence of –CH(7′)–CH(8′)–CH_2_(9′)– fragment, also supported by HMBC correlations of *δ*_H_ 4.88 (1H, d, *J* = 8.1 Hz, H-7′), 4.07 (1H, ddd, *J* = 8.1, 4.3, 2.5 Hz, H-8′) to *δ*_C_ 106.0 (d, C-2′, 6′) (Fig. 2). The relative configuration of H-7′/H-8′ was *threo* orientation, for the large coupling constant (*J* = 8.1 Hz) between H-7′ and H-8′, along with ROESY correlation of H-2′ and H-6′ with H-8′ and there was no NOE correlation between H-7′ and H-8′ [33–35] (Fig. 3). Other parts of the structure were identical to those of **9** by detailed analysis of its 2D NMR spectra. Thus, compound **4** was assigned as (*Z*)-thoreliamide B.

A literature survey shows that the *E*-isomers of this type of compounds are widespread in some genera and small amount of *Z*-isomers [12, 18]. In this paper, three pairs of Z-*E*-isomers (**3** and **6**; **4** and **9**; **7** and **8**) were reported. Due to their different retention times on the Rp-C_18_ column, every pair of isomers was separated by HPLC.

**Fig. 4:**
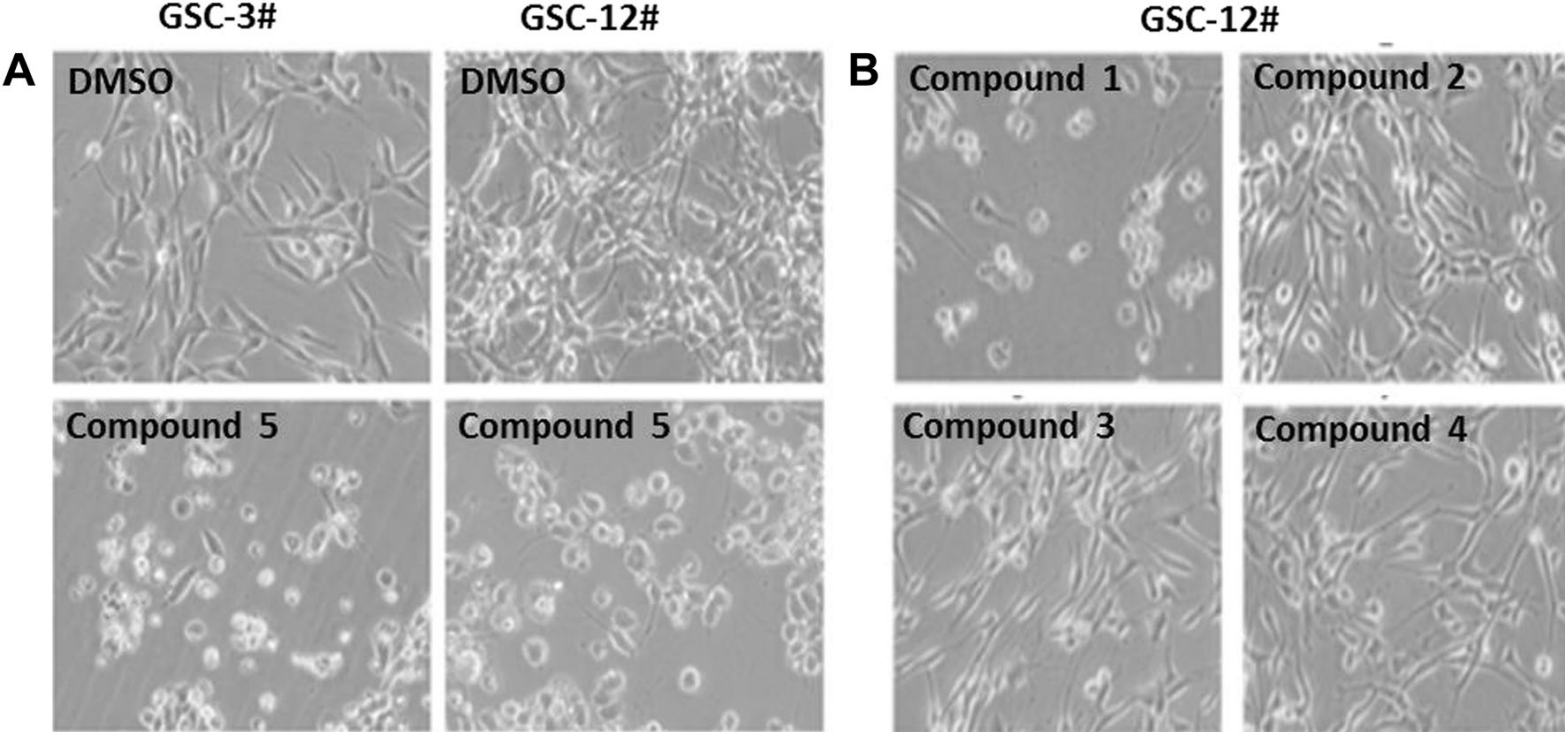
Compounds **1**–**5** against human glioma stem cells by phenotypic screening; **a** Compound **5** against GSC-3# and GSC-12# at 10 μg/mL; **b** Compounds **1**–**4** against GSC-12# at 25 μg/mL

**Fig. 5:**
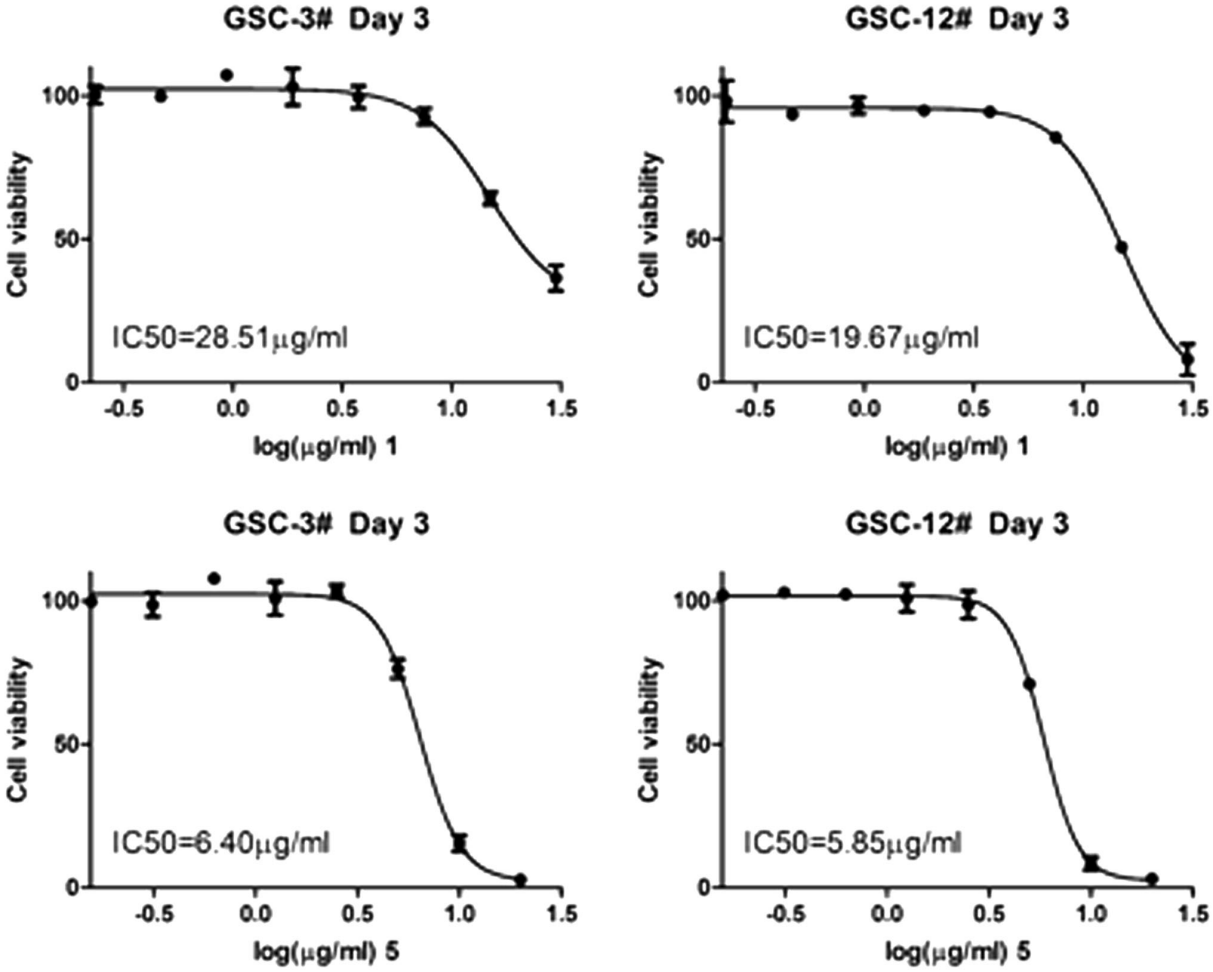
The IC_50_ value for compounds **1** and **5** against human glioma stem cell lines

All the compounds were evaluated for their bioactivity against two human glioma stem cell lines (GSC-3# and GSC-12#), by the cell viability assay and phenotypic screening. The results showed that compound **5** exhibited the moderate cytotoxicity against GSC-3# and GSC-12# at the concentration of 10 μg/mL (Fig. 4a), and the IC_50_ values were 6.40 and 5.85 μg/mL respectively (Fig. 5). Compound **1** showed the moderate cytotoxicity against GSC-3# and GSC-12# at the concentration of 25 μg/mL (Fig. 4b), and the IC_50_ values were 28.51 and 19.67 μg/mL respectively (Fig. 5).

## Experimental Section

### General Experimental Procedures

Optical rotations were measured on a JASCO P-1020 polarimeter. UV spectra were detected on a SHMADZU UV-2401PC spectrometer. IR spectra were determined on a Bruker FT-IR Tensor-27 infrared spectrophotometer with KBr disks. 1D and 2D NMR spectra were recorded on Bruker DRX-400, DRX-500, and DRX-600 spectrometers using TMS as an internal standard. Chemical shifts (*δ*) were expressed in ppm with reference to the solvent signals. ESI–MS and EI–MS (HR–EI–MS) analysis were carried out on Waters Xevo TQS and Waters AutoSpec Premier P776 mass spectrometers, respectively. Semi-preparative HPLC was performed on a Waters 600 with a COSMOSIL C_18_ (10 × 250 mm) column. Silica gel (100–200 and 200–300 mesh, Qingdao Marine Chemical Co., Ltd., People’s Republic of China), and MCI gel (75–150 μm, Mitsubishi Chemical Corporation, Tokyo, Japan) were used for column chromatography. Fractions were monitored by thin-layer chromatography (TLC) (GF254, Qingdao Marine Chemical Co., Ltd.), and spots were visualized by 10% sulfuric acid ethanol solution and dragendorff reagent.

### Plant Material

The stem of *L. barbarum* were collected from Zhongning county of Ningxia province, where the Chinese wolfberries producing, and identified by Mr. Jianfei Liu, Lanzhou Institute of Chemical Physics, CAS. A voucher specimen (No. 20150602) has been deposited in the Kunming Institute of Botany, Chinese Academy of Sciences, Kunming, China.

### Extraction and Isolation

The air-dried and powdered sample (10.0 kg) was extracted with 85% aqueous EtOH (40 L × 3) under reflux conditions for 3 h each time, and the solvent was evaporated in vacuum. The residue (607 g) was suspended in H_2_O and extracted with EtOAc (each 2 L × 3). The EtOAc layer (224 g) was passed over a silica gel column, eluting with CHCl_3_–Me_2_CO (1:0 to 0:1) to give nine fractions. Fraction VI (22 g) was chromatographed on silica gel, eluted with CHCl_3_–MeOH (20:1 to 6:1) to give **12** (568.9 mg) and the residue, then the latter was further purified over HPLC to afford **2** (4.86 mg, *t*_R_ 18 min; CH_3_CN/H_2_O 20:80, 3 mL/min), and **11** (15.5 mg, *t*_R_ 23.5 min; CH_3_CN/H_2_O 20:80, 3 mL/min). Fraction VII (62 g) was separated on a MCI column eluted successively with MeOH/H_2_O (10:1–5:1) to afford seven subfractions VII-1–7 and one white needle crystal **16** (7283.1 mg, in chloroform). Subfraction VII-2 was separated by Sephadex LH-20 column, eluted with MeOH, to afford compound **14** (1154.4 mg). Subfraction VII-3 was separated by RP-MPLC, eluted with MeOH/H_2_O (20:100–55:100) to get subfractions VII-3-1 and VII-3-2. Subfraction VII-3-1 was chromatographed on silica gel, eluted with CHCl_3_–MeOH (20:1–5:1) to give **15** (535.7 mg), **17** (88.0 mg) and **13** (168.3 mg). Subfraction VII-3-2 was purified over HPLC to afford **10** (23.1 mg, *t*_R_ 28 min; CH_3_CN/H_2_O 28:72, 3 mL/min). Subfraction VII-4 was separated on the column of polyamide, eluted with MeOH/H_2_O (45:100–60:100), to yield compound **5** (185.0 mg) and a mixture. Further separation of the mixture by on a silica gel column (CHCl_3_/MeOH, 20:1–9:1) yielded compound **1** (43.5 mg). Subraction VII-5 firstly separated by Sephadex LH-20 column to obtain two major subfraction VII-5-1 and VII-5-2, and the former was purified by HPLC to afford **4** (4.2 mg, *t*_R_ 16 min; CH_3_CN/H_2_O 35:65, 3 mL/min) and **9** (5.6 mg, *t*_R_ 21.5 min; CH_3_CN/H_2_O 35:65, 3 mL/min). Separation of subfraction VII-5-2 by silica gel column (CHCl_3_/MeOH, 20:1–9:1) to obtain subfractions VII-5-2a and VII-5-2b. Purified of subfraction VII-5-2a by HPLC afford **6** (54.3 mg, *t*_R_ 18 min; CH_3_CN/H_2_O 38:62, 3 mL/min), **3** (43.2 mg, *t*_R_ 23 min; CH_3_CN/H_2_O 38:62, 3 mL/min). Subfraction VII-5-2b was separated by Sephadex LH-20 column (elution with MeOH), and then chromatographed on silica gel column (CHCl_3_/MeOH, 10:1), further purified by HPLC to afford **7** (12.5 mg, *t*_R_ 20 min; CH_3_CN/H_2_O 38:62, 3 mL/min) and **8** (7.8 mg, *t*_R_ 25 min; CH_3_CN/H_2_O 38:62, 3 mL/min).

#### 4-O-methylgrossamide (**1**)

White powder; $[\upalpha]_{\text{D}}^{25}$ − 16.8° (c 0.11, DMSO); UV (MeOH) *λ*_max_ (log *ε*) 249 (4.2), 289 (4.4), 320(4.4); IR (KBr) *ν*_max_ 3387, 1651, 1605, 1517, 1265 cm^−1^; ^1^H NMR (DMSO**-** *d*_6_, 600 MHz) and ^13^C NMR (DMSO**-** *d*_6_, 150 MHz) data, see Table 1; positive ESIMS m/z 639 [M+H]^+^; positive HRESIMS m/z 639.2721 [M+H]^+^ (calcd. for C_37_H_39_N_2_O_8_, 639.2701).

#### (E)-2-(4,5-dihydroxy-2-{3-[(4-hydroxyphenethyl)amino]-3-oxopro-pyl}phenyl)-3-(4-hydroxy-3-methoxyphenyl)-N-(4-hydroxyphenethyl)-acrylamide (**2**)

Yellow powder; $[\upalpha]_{\text{D}}^{19}$ − 2.3° (c 0.47, MeOH); UV (MeOH) *λ*_max_(log *ε*) 203 (4.7), 217 (4.6), 289 (4.1), 324 (4.1); IR (KBr) *ν*_max_ 3422, 2927, 1636, 1614, 1514, 1449, 1262 cm^−1^; ^1^H NMR (DMSO**-** *d*_6_, 600 MHz) and ^13^C NMR (DMSO**-** *d*_6_, 150 MHz) data, see Table 1; positive HRESIMS [M+Na]^+^ 635.2368 (calcd. for C_35_H_36_N_2_O_8_Na, 635.2364).

#### Z-lyciumamide C (**3**)

White powder; $[\upalpha]_{\text{D}}^{26}$ − 5.0° (c 0.11, MeOH); UV (MeOH) *λ*_max_ (log *ε*) 203 (4.6), 225 (4.5), 286 (4.2), 304 (4.2); IR (KBr) *ν*_max_ 3408, 1647, 1608, 1516, 1273, 1216 cm^−1^; ^1^H NMR (CD_3_OD, 500 MHz) and ^13^C NMR (CD_3_OD, 125 MHz) data, see Table 2; positive ESIMS m/z 492 [M+H]^+^; positive HRESIMS m/z 492.2027 [M+H]^+^ (calcd. for C_28_H_30_NO_7_, 492.2017).

#### Z-thoreliamide B (4)

Faint yellow powder; $[\upalpha]_{\text{D}}^{21}$ − 11.1° (c 0.11, MeOH); UV (MeOH) *λ*_max_ (log *ε*) 207 (4.7), 240 (4.3), 278 (4.0); IR (KBr) *ν*_max_ 3421, 1648, 1614, 1512, 1274, 1219 cm^−1^; ^1^H NMR (CD_3_OD, 600 MHz) and ^13^C NMR (CD_3_OD, 150 MHz) data, see Table 1; positive ESIMS m/z 508 [M+H]^+^; negtive HRESIMS m/z 506.1825 [M−H]^−^ (calcd. for C_28_H_28_NO_8_, 506.1820).

### Anticancer Activities

GSC-3# and GSC-12# were human glioma stem cell lines that were established by Kunming institute of zoology from two human glioblastoma multiform samples. The glioma stem cell was cultured in serum-free medium DMEM F12 supplied with 1xB27 and 50 ng/mL EGF, BFGF and 1% penicillin/streptomycin. The glioma stem cells were seeded in the laminin pre-coating dishes and cultured in 37 °C, 5% CO_2_ incubator. Cell viability assay was performed by the MTS method as previously described. GSCs were digested and counted, seeded in laminin pre-coating 96-well-plate with 20000 cells/well. The compounds were added with a serial gradient concentration (40, 20, 10, 5, 2.5, 1.25, 0.625, 0.3125 μg/mL) and cultured in cell incubator for 72 h. MTS reagent was diluted 1:5 with fresh medium and mixed well. The old medium was removed and subsequently the fresh medium was added with 100 μL/well. The cells were incubated for 1.5 h. Absorbance was measured by Hybrid Reader (BioTek synergy H1) at 490 nm. The cell viability was evaluated by percentage compared with DMSO control group. The half-maximal inhibitory concentration (IC_50_) was measured and calculated by Graph Pad Prism 5 software.

## Supporting Information

1D, 2D NMR spectra, ESIMS/MS, HRESIMS, UV and IR of compounds **1**–**4** and influence of deuterated solvent on the ^1^H NMR spectra of compounds **3** and **6** are available).

## Electronic supplementary material

Below is the link to the electronic supplementary material.

Supplementary material 1 (PDF 4735 kb)
