# Supplementary material for: The Anticancer Activities Phenolic Amides from the Stem of Lycium barbarum
Source: Nat Prod Bioprospect. 2017 Jun 6;7(6):421–31. doi: 10.1007/s13659-017-0134-x (PMC5709248; doi:10.1007/s13659-017-0134-x)
Supplement: Supplementary file 1 — Supplementary material 1 (PDF 4735 kb) [file 13659_2017_134_MOESM1_ESM.pdf]

Supplementary Information

The Anticancer Activities Phenolic Amides from the Stem of *Lycium barbarum*

Pei-Feng Zhu,[a,b,c] Zhi Dai,[b,d] Bei Wang,[a,b,c] Xin Wei,[a,b,c] Hao-Fei Yu,[a,b,c] Zi-Ru Yan,[a,b,c] Xu-Dong Zhao,[d] Ya-Ping Liu,[a,c,*] and Xiao-Dong Luo[a,c,*]

[a] *State Key Laboratory of Phytochemistry and Plant Resources in West China, Kunming Institute of Botany, Chinese Academy of Sciences, Kunming 650201, P. R. China*

[b] *University of Chinese Academy of Sciences, Beijing 100049, People's Republic of China*

[c] *Yunnan Key Laboratory of Natural Medicinal Chemistry, Kunming, 650201, People's Republic of China*

[d] *Key Laboratory of Animal Models and Human Disease Mechanisms of Chinese Academy of Sciences/Key Laboratory of Bioactive Peptides of Yunnan Province, Kunming Institute of Zoology, Kunming 650223, Yunnan, China*

[*] **Corresponding author** Prof. Dr. Xiao-Dong Luo, Dr. Ya-Ping Liu,

E-mail: xdluo@mail.kib.ac.cn (X. D. Luo); liuyaping@mail.kib.ac.cn (Y. P. Liu)

Supporting Information

Figure S1. ^{1}H NMR spectrum of compound **1**

Figure S2. ^{13}C NMR spectrum of compound **1**

Figure S3. HSQC spectrum of compound **1**

Figure S4. HMBC spectrum of compound **1**

Figure S5. COSY spectrum of compound **1**

Figure S6. ROESY spectrum of compound **1**

Figure S7. ESIMS spectrum of compound **1**

Figure S8. ESIMS/MS spectrum of compound **1**

Figure S9. HRESIMS spectrum of compound **1**

Figure S10. UV spectrum of compound **1**

Figure S11. IR spectrum of compound **1**

Figure S12. Optical rotation of compound **1**

Figure S13. Circular dichroic spectrum of compound **1**

Figure S14. ^{1}H NMR spectrum of compound **2**

Figure S15. ^{13}C NMR spectrum of compound **2**

Figure S16. HSQC spectrum of compound **2**

Figure S17. HMBC spectrum of compound **2**

Figure S18. COSY spectrum of compound **2**

Figure S19. ROESY spectrum of compound **2**

Figure S20. ESIMS spectrum of compound **2**

Figure S21. ESIMS/MS spectrum of compound **2**

Figure S22. HRESIMS spectrum of compound **2**

Figure S23. UV spectrum of compound **2**

Figure S24. IR spectrum of compound **2**

Figure S25. Optical rotation of compound **2**

Figure S26. ^{1}H NMR spectrum of compound **3**

Figure S27. ^{13}C NMR spectrum of compound **3**

Figure S28. HSQC spectrum of compound **3**

Figure S29. HMBC spectrum of compound **3**

Figure S30. COSY spectrum of compound **3**

Figure S31. ROESY spectrum of compound **3**

Figure S32. ESIMS spectrum of compound **3**

Figure S33. HRESIMS spectrum of compound **3**

Figure S34. UV spectrum of compound **3**

Figure S35. IR spectrum of compound **3**

Figure S36. Optical rotation of compound **3**

Figure S37. Influence of deuterated solvent on the ^{1}H NMR spectra of compound **3**

Figure S38. Influence of deuterated solvent on the ^{1}H NMR spectra of compound **6**

Figure S39. ^{1}H NMR spectrum of compound **4**

Figure S40. ^{13}C NMR spectrum of compound **4**

Figure S41. HSQC spectrum of compound **4**

Figure S42. HMBC spectrum of compound **4**

Figure S43. COSY spectrum of compound **4**

Figure S44. ROESY spectrum of compound **4**

Figure S45. ESIMS spectrum of compound **4**

Figure S46. ESIMS/MS spectrum of compound **4**

Figure S47. HRESIMS spectrum of compound **4**

Figure S48. UV spectrum of compound **4**

Figure S49. IR spectrum of compound **4**

Figure S50. Optical rotation of compound **4**

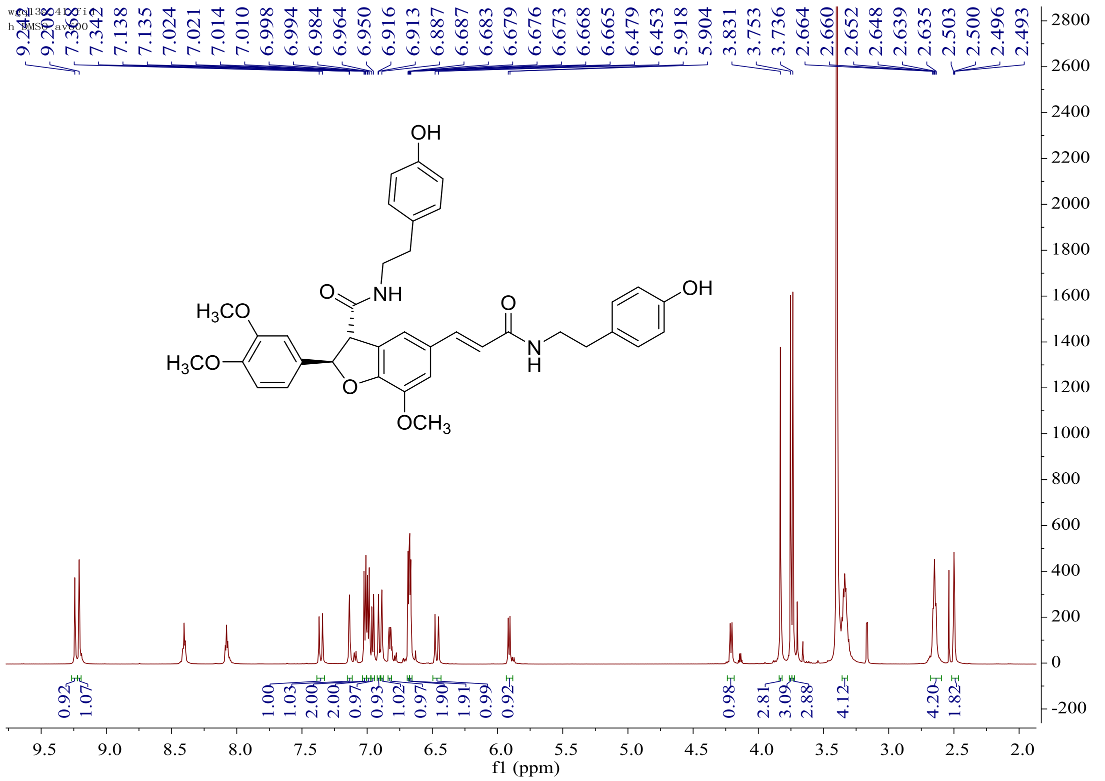

Figure S1.^{1}H NMR spectrum of compound **1**(DMSO-d_6, 600MHz)

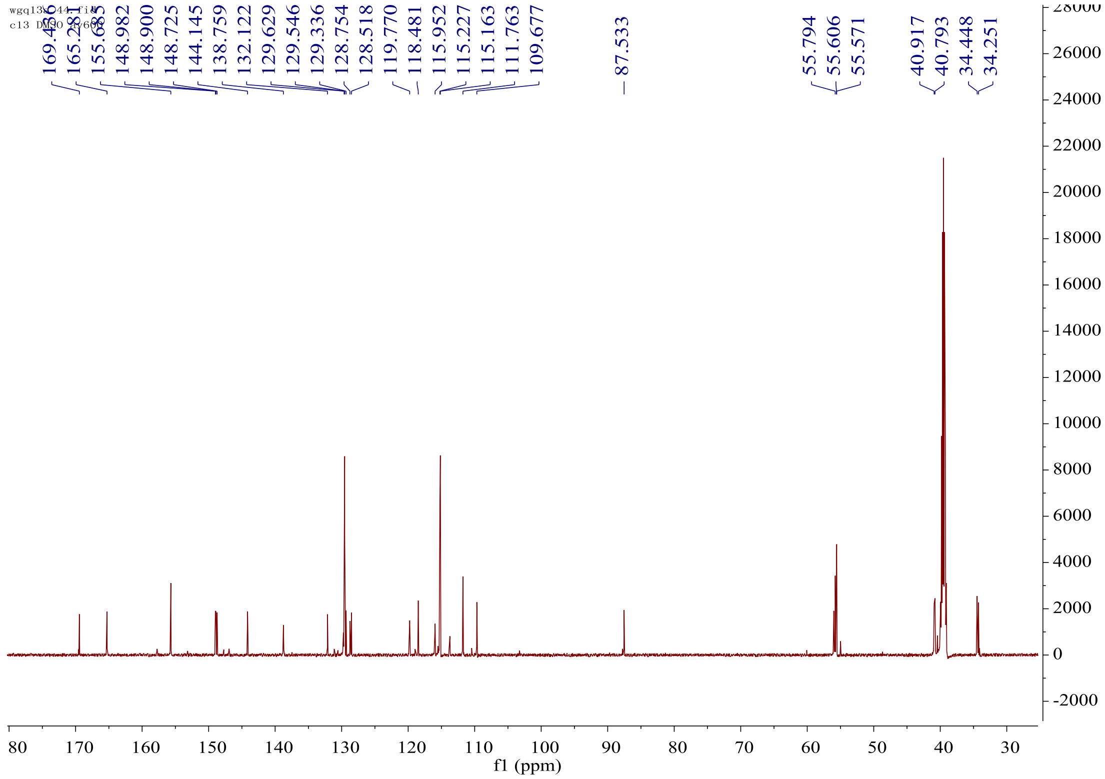

Figure S2.^{13}C NMR spectrum of compound **1**(DMSO-d_6, 150MHz)

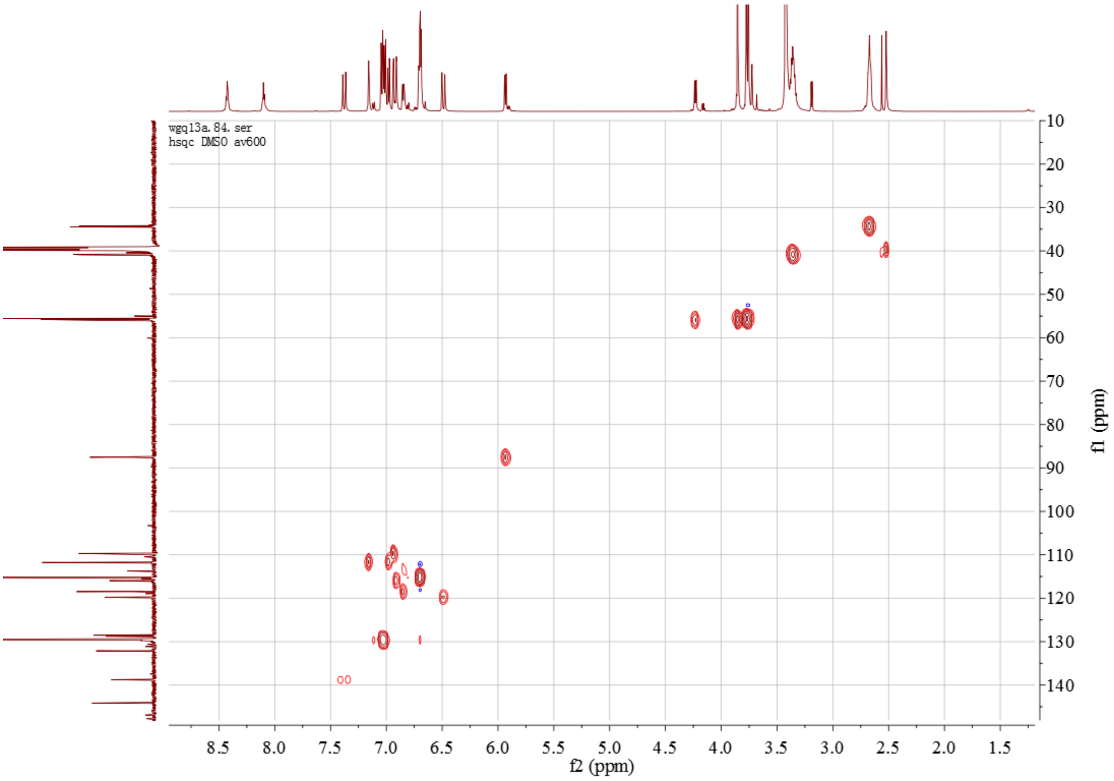

Figure S3.HSQC spectrum of compound **1**

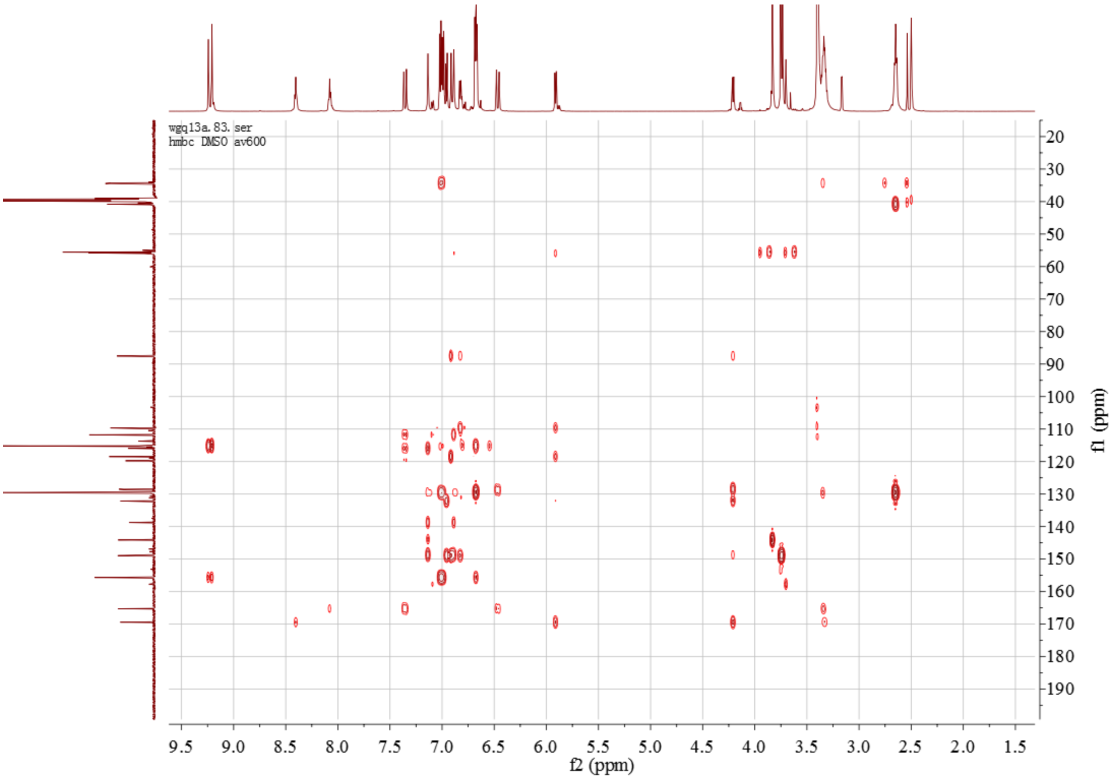

Figure S4.HMBC spectrum of compound **1**

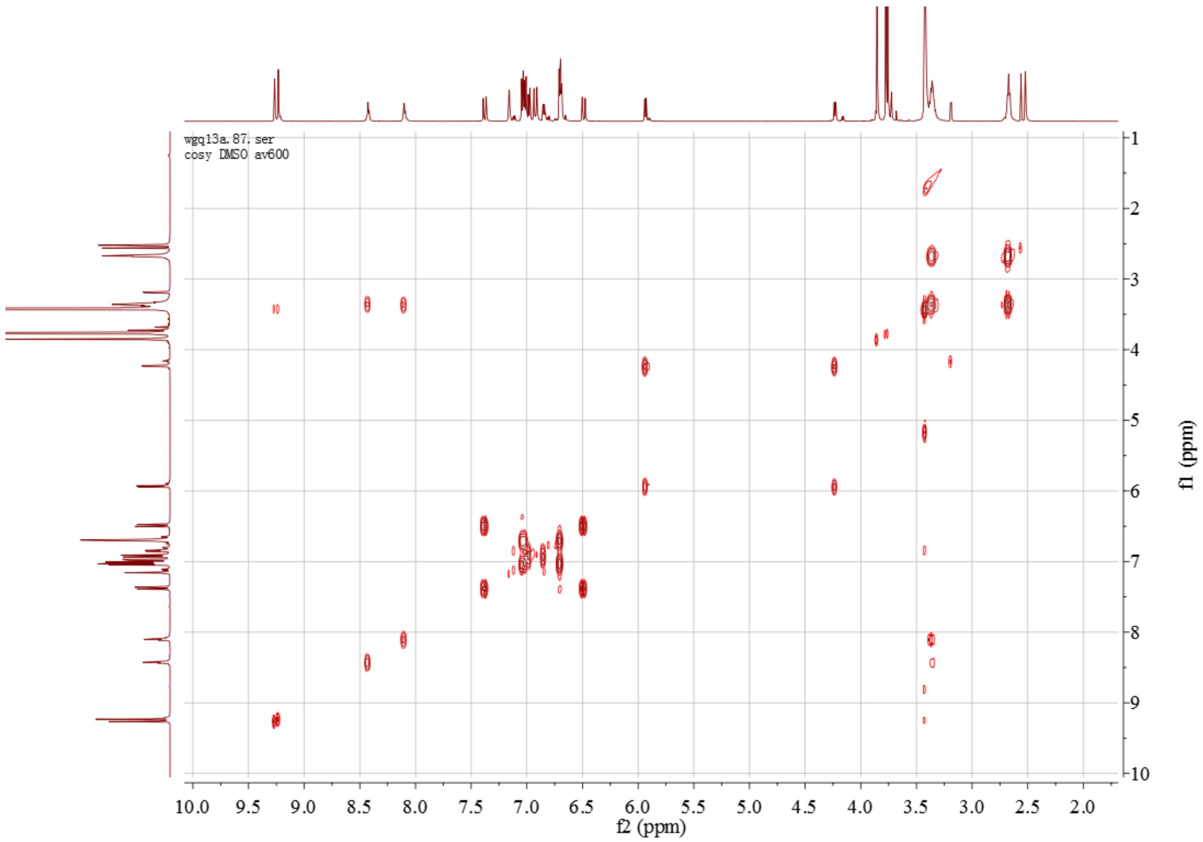

Figure S5.COSY spectrum of compound **1**

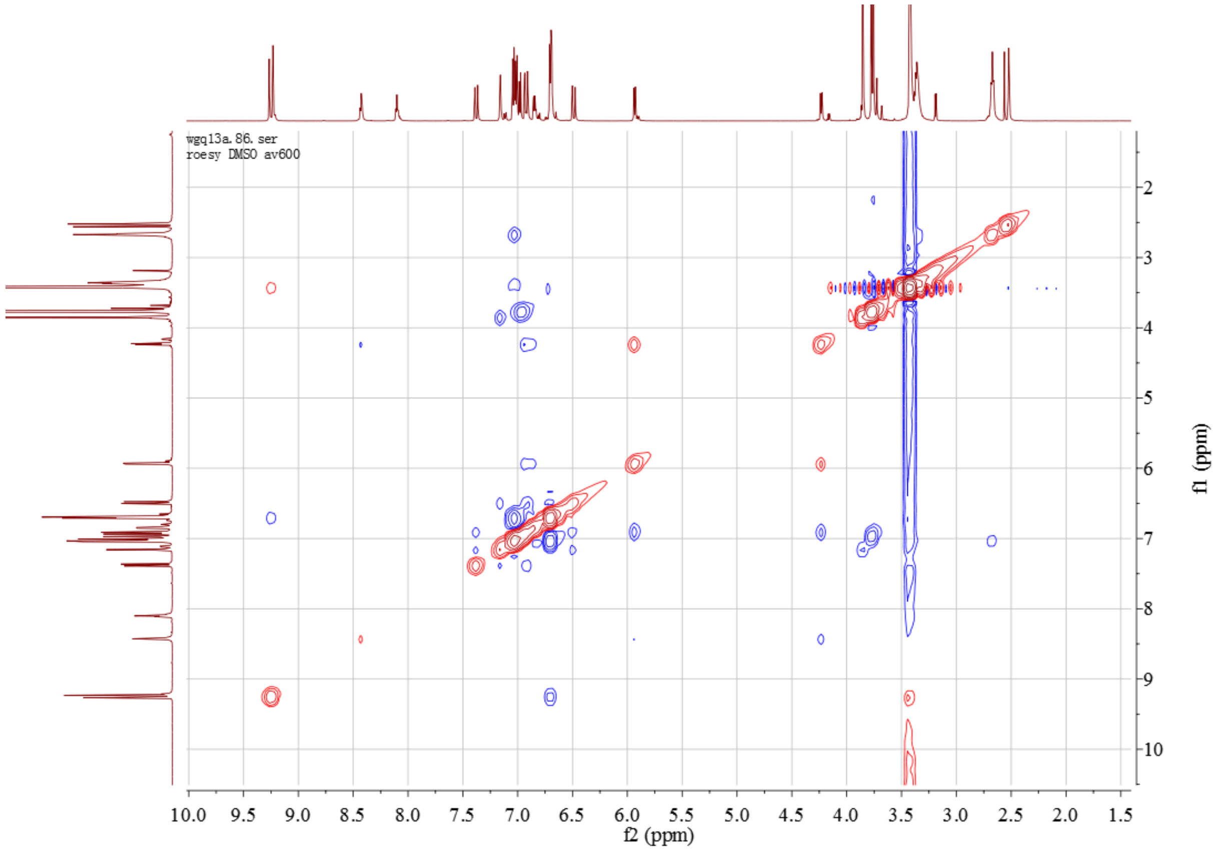

Figure S6.ROESY spectrum of compound **1**

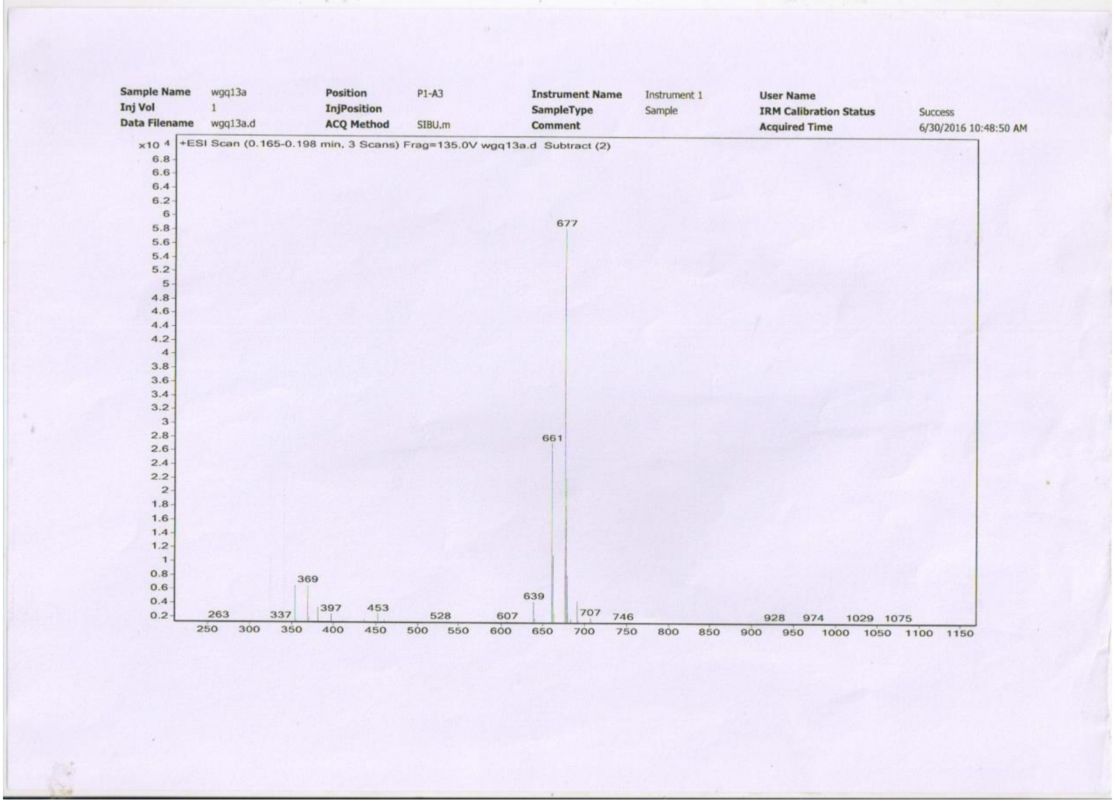

Figure S7. ESIMS spectrum of compound **1**

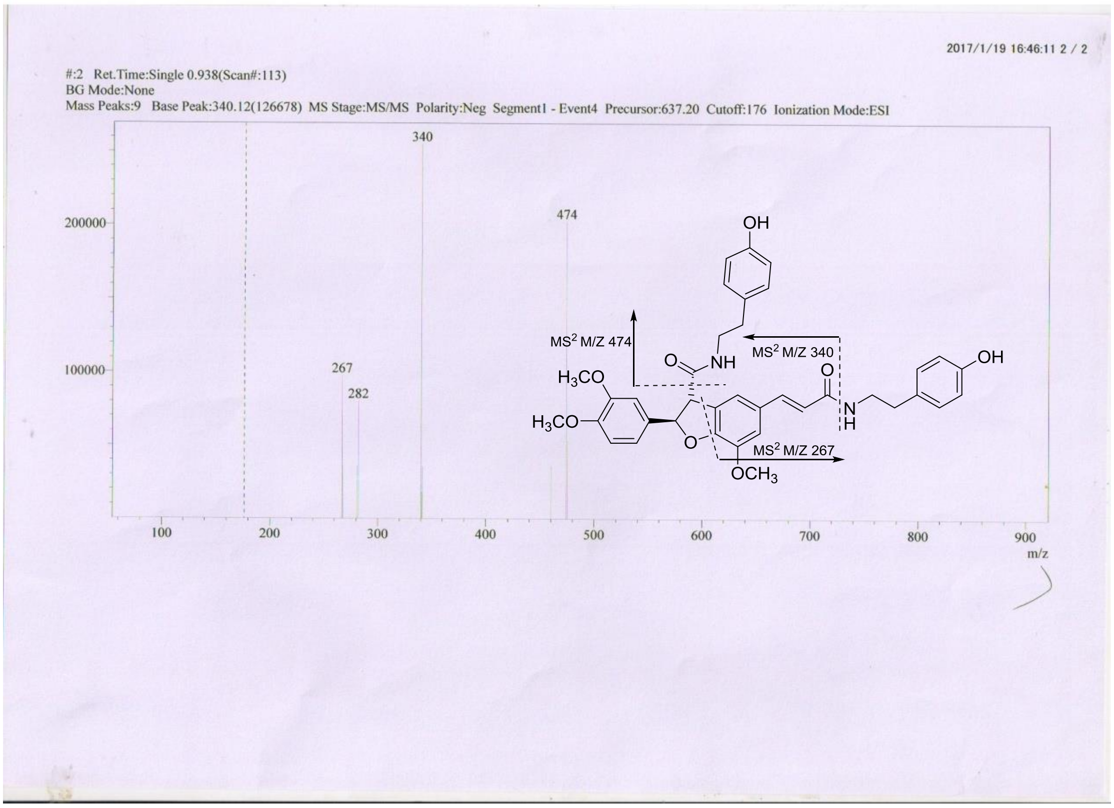

Figure S8. ESIMS/MS spectrum of compound **1**

Qualitative Analysis Report

**Data Filename**	wgq13a.d	**Sample Name**	wgq13a
**Sample Type**	Sample	**Position**	P1-C3
**Instrument Name**	Instrument 1	**User Name**	
**Acq Method**	SIBU.m	**Acquired Time**	10/18/2016 3:50:49 PM
**IRM Calibration Status**	Success	**DA Method**	ESI+.m
**Comment**			

Sample Group **Info.**
Acquisition SW Version 6200 series TOF/6500 series Q-TOF B.05.01 (B5125.2)

User Spectra

Fragmentor Voltage	Collision Energy	Ionization Mode
175	0	ESI

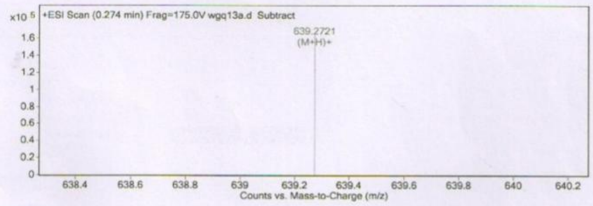

Peak List

m/z	z	Abund	Formula	Ion
339.1107	2	21891.04		
639.2721	1	166575.63	C37 H38 N2 O8	(M+H)+
640.2747	1	62110.08	C37 H38 N2 O8	(M+H)+
641.2766	1	13321.66	C37 H38 N2 O8	(M+H)+
661.2534	1	38598.01		
662.2552	1	16098.02		
677.2263	1	20203.58		
1277.5393	1	10479.07		

Formula Calculator Element Limits

Element	Min	Max
C	3	60
H	0	120
O	0	30
N	0	5

Formula Calculator Results

Formula	CalculatedMass	CalculatedMz	Mz	Diff. (mDa)	Diff. (ppm)	DBE
C37 H38 N2 O8	638.2628	639.2701	639.2721	-1.7	-2.7	20.0000

--- End Of Report ---

Figure S9. HRESIMS spectrum of compound **1**

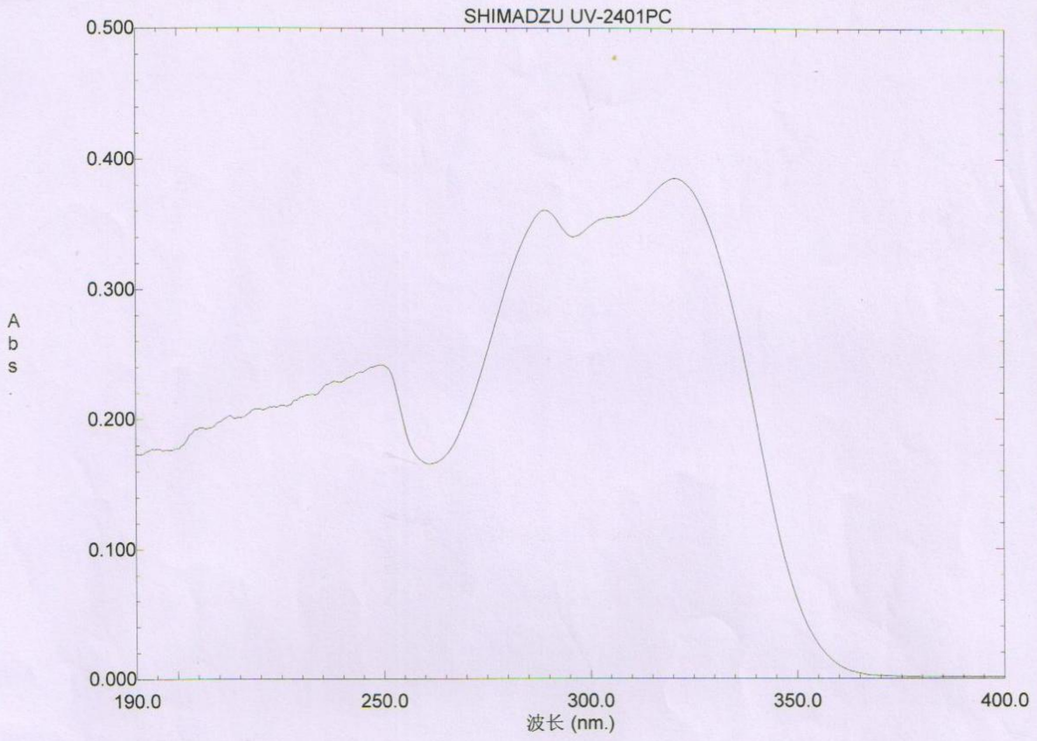

文件名:　WGQ13A

WGQ13A

样品浓度：0.0101毫克/毫升
溶剂：DMSO

创建于:　12:14　16-10-14
数据:　原始

测量模式:　Abs.
扫描速度:　中速
狭缝:　5.0
采样间隔:　0.5

否.	波长 (nm.)	Abs.
1	320.50	0.3847
2	289.00	0.3603
3	249.00	0.2414

Figure S10. UV spectrum of compound **1**

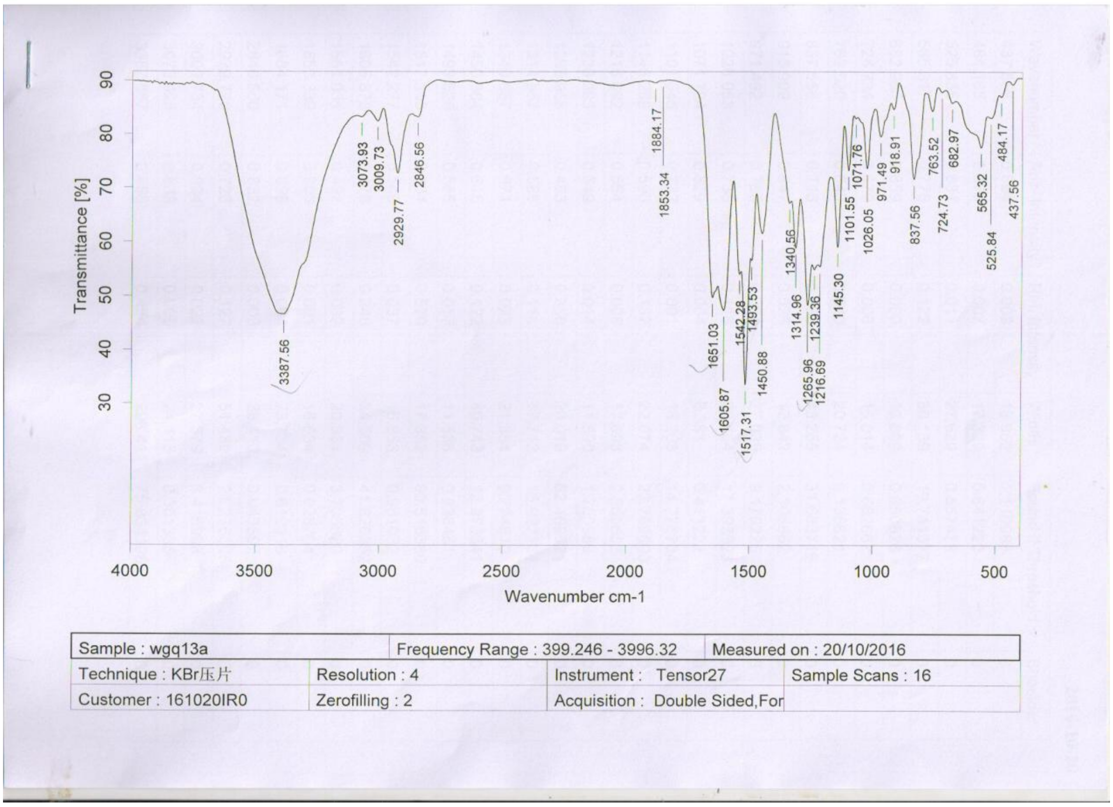

Figure S11. IR spectrum of compound **1**

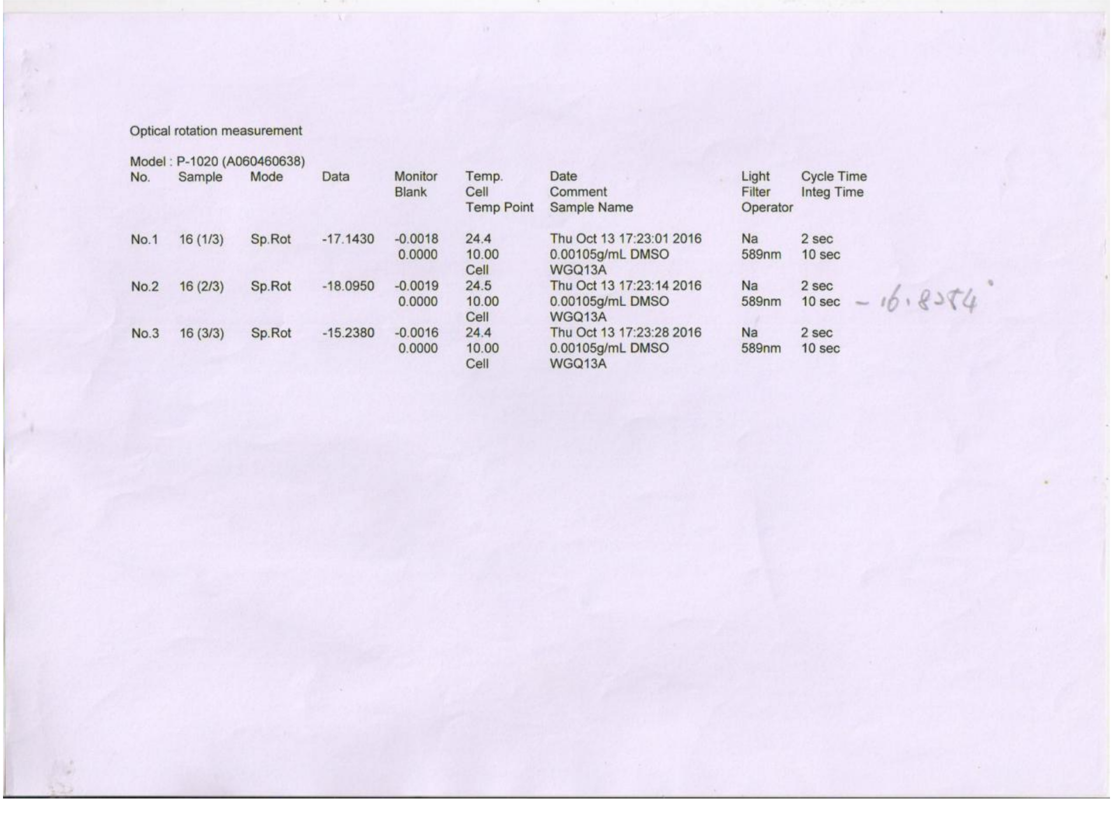

Optical rotation measurement

Model : P-1020 (A060460638)

No.	Sample	Mode	Data	Monitor / Blank	Temp. / Cell / Temp Point	Date / Comment / Sample Name	Light / Filter / Operator	Cycle Time / Integ Time
No.1	16 (1/3)	Sp.Rot	-17.1430	-0.0018 / 0.0000	24.4 / 10.00 / Cell	Thu Oct 13 17:23:01 2016 / 0.00105g/mL DMSO / WGQ13A	Na / 589nm	2 sec / 10 sec
No.2	16 (2/3)	Sp.Rot	-18.0950	-0.0019 / 0.0000	24.5 / 10.00 / Cell	Thu Oct 13 17:23:14 2016 / 0.00105g/mL DMSO / WGQ13A	Na / 589nm	2 sec / 10 sec — 16.8254°
No.3	16 (3/3)	Sp.Rot	-15.2380	-0.0016 / 0.0000	24.4 / 10.00 / Cell	Thu Oct 13 17:23:28 2016 / 0.00105g/mL DMSO / WGQ13A	Na / 589nm	2 sec / 10 sec

Figure S12. Optical rotation of compound **1**

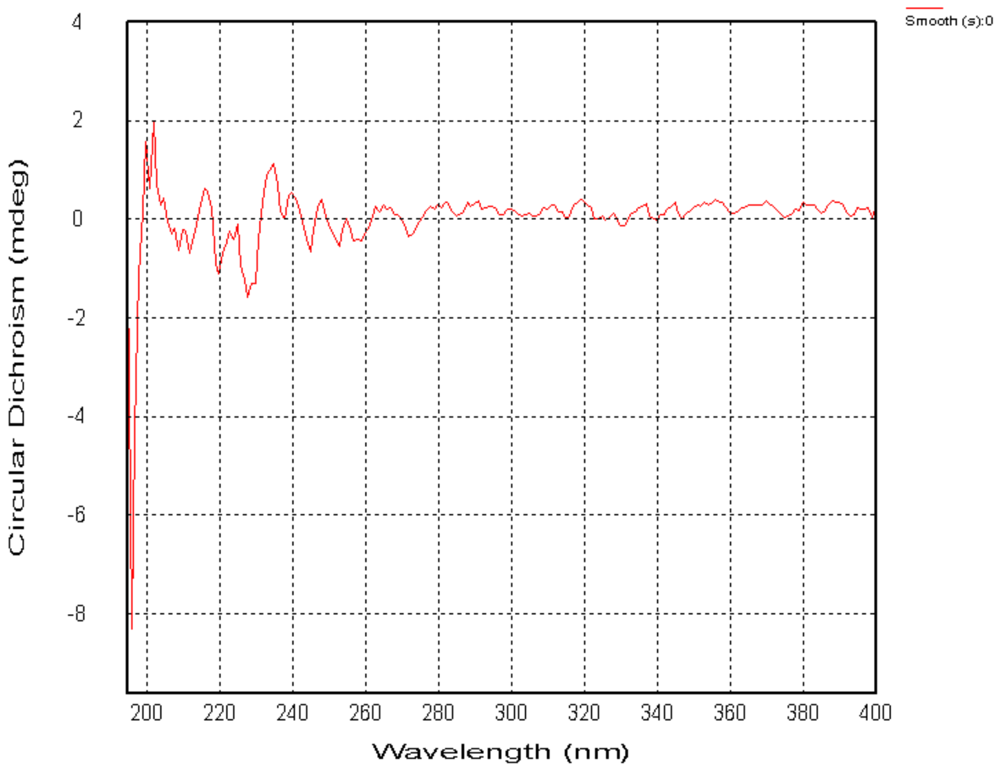

Figure S13. Circular dichroic spectrum of compound **1**

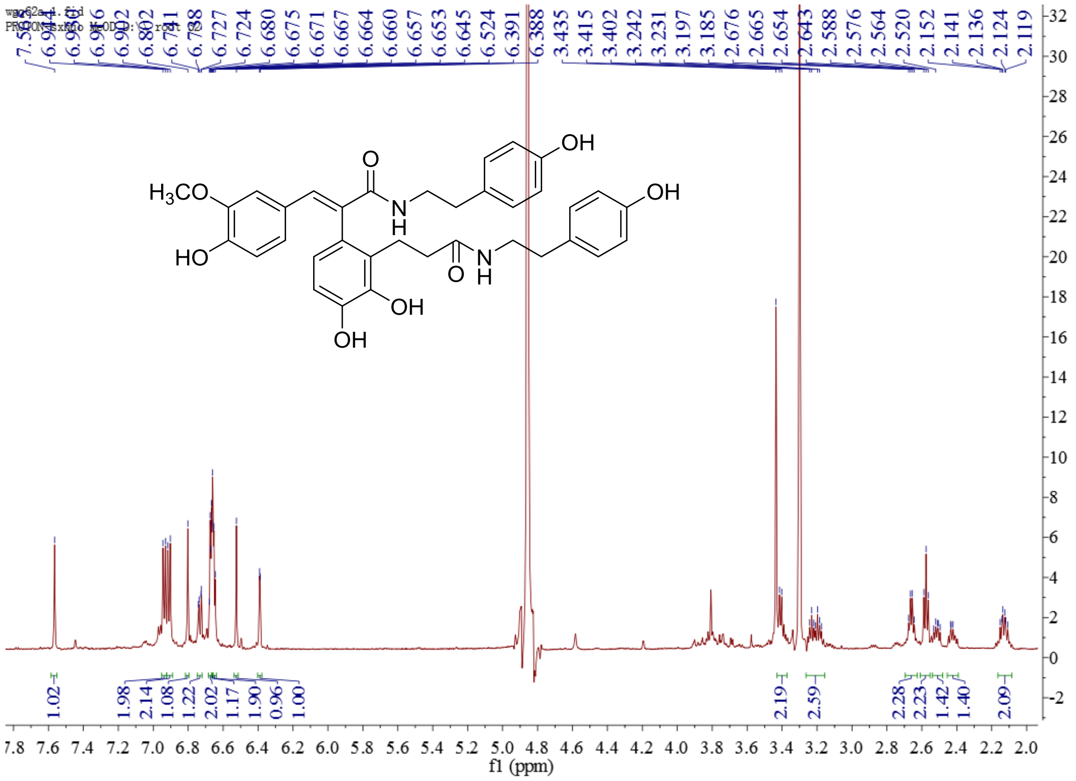

Figure S14.^{1}H NMR spectrum of compound **2**

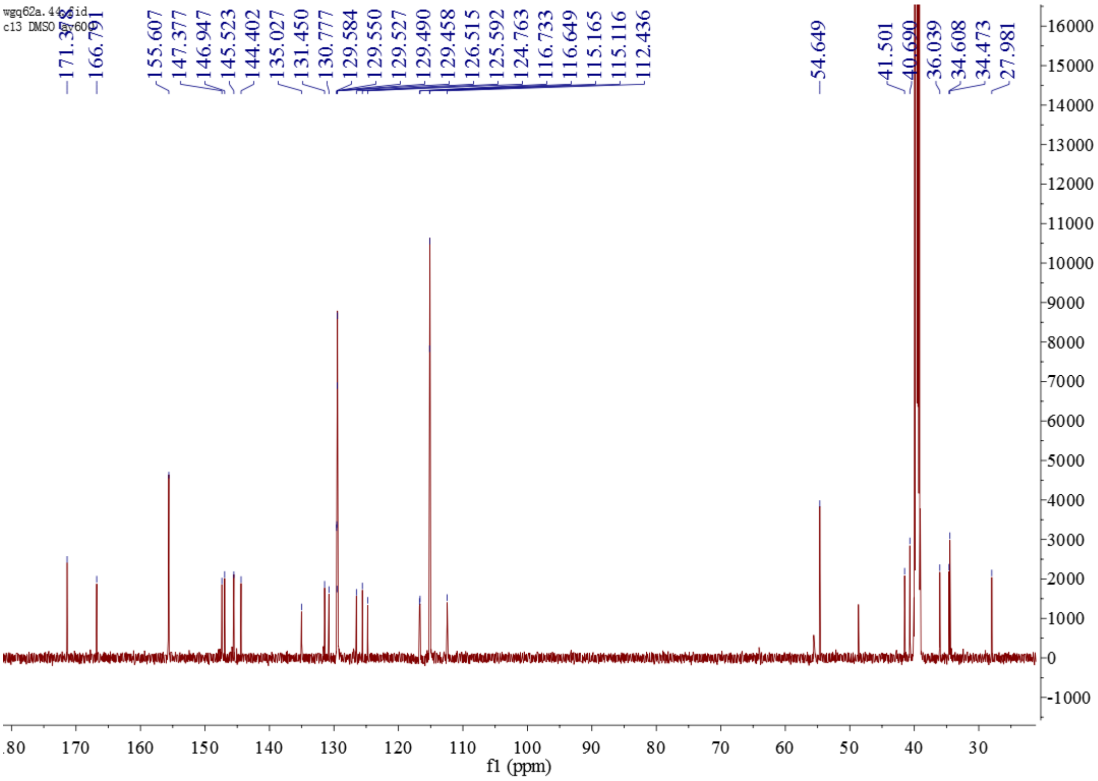

Figure S15.^{13}C NMR spectrum of compound **2**

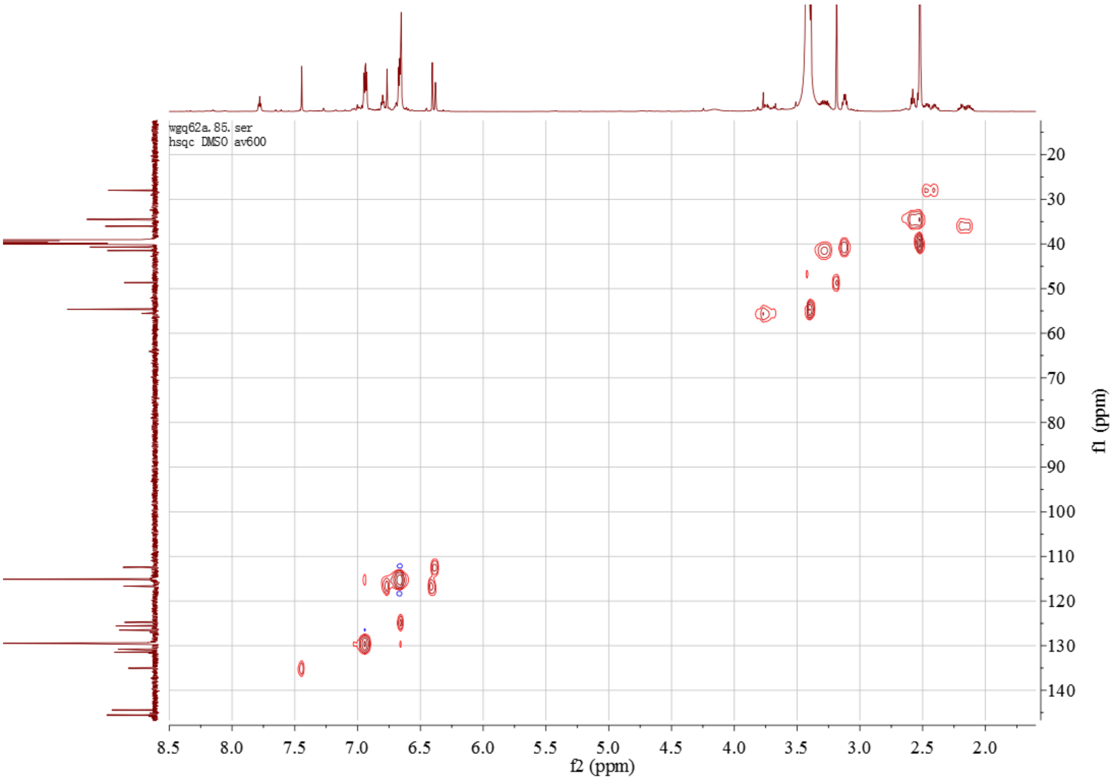

Figure S16.HSQC spectrum of compound **2**

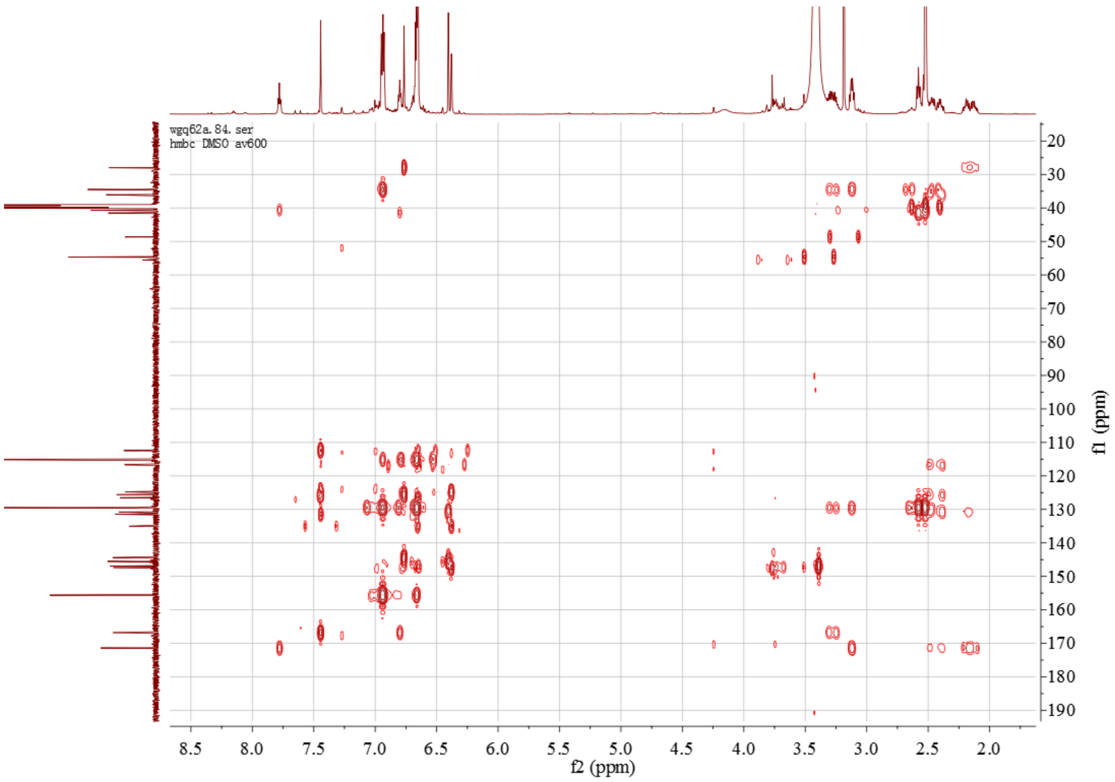

Figure S17. HMBC spectrum of compound **2**

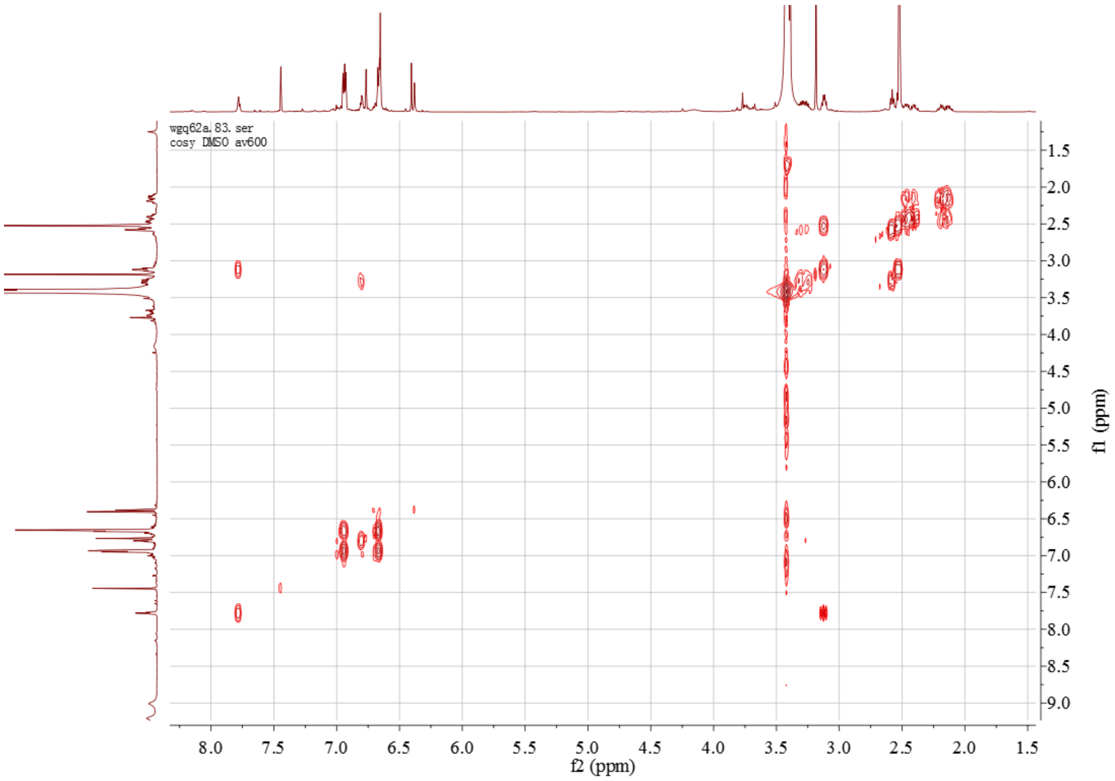

Figure S18. COSY spectrum of compound **2**

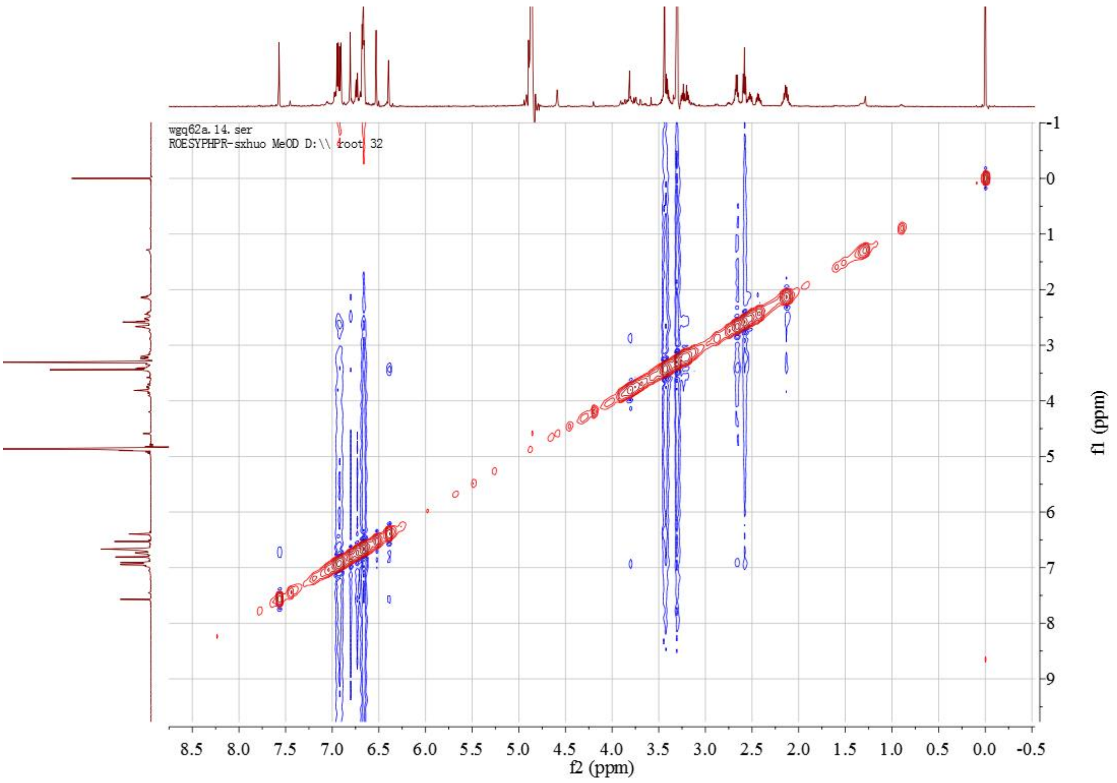

Figure S19. ROESY spectrum of compound **2**

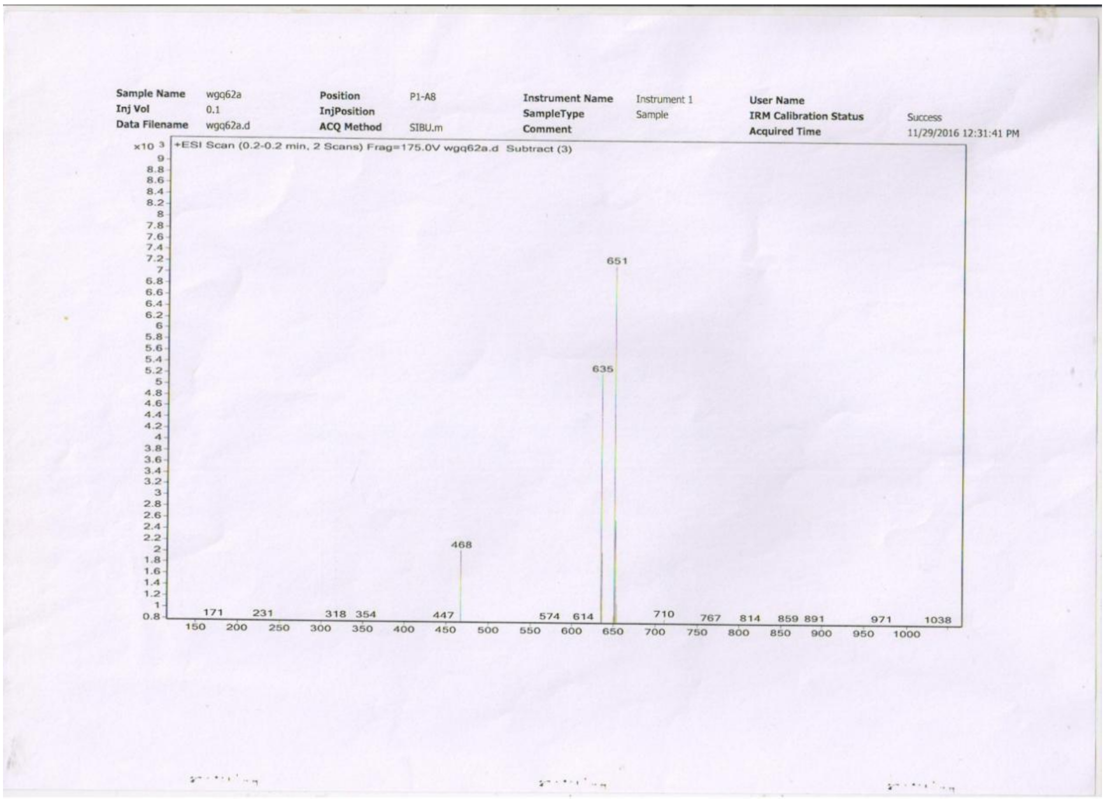

Figure S20. ESIMS spectrum of compound **2**

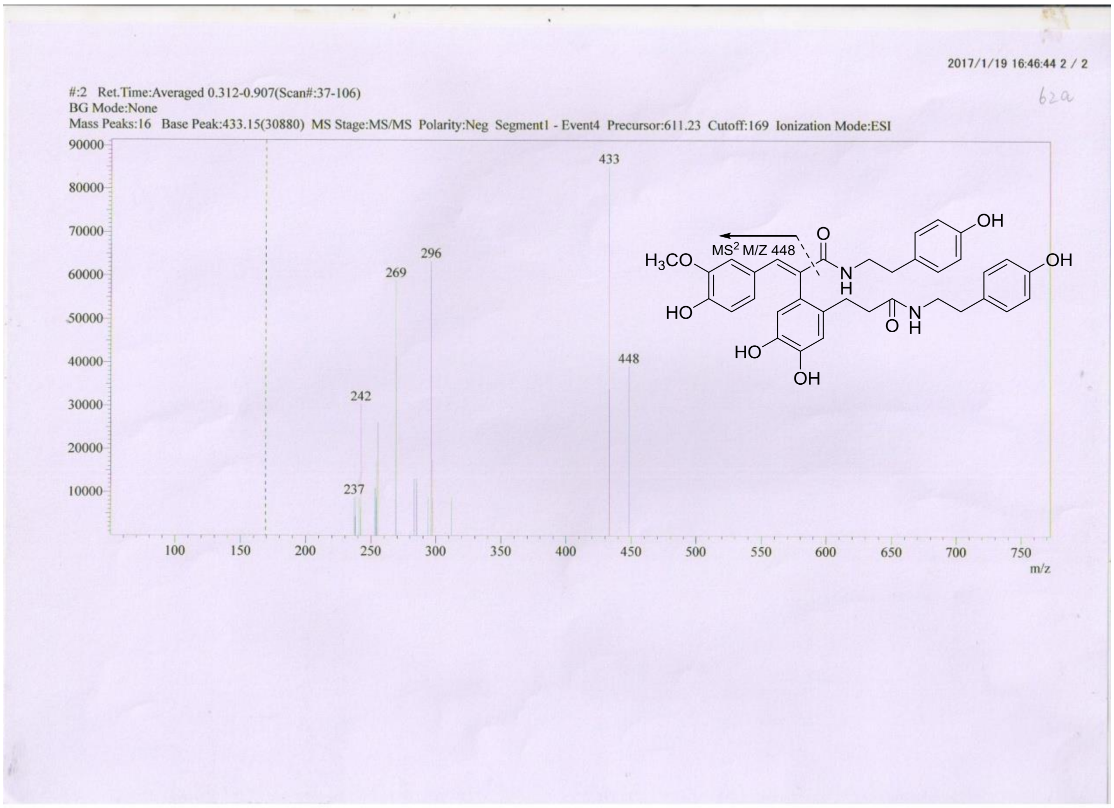

Figure S21. ESIMS/MS spectrum of compound **2**

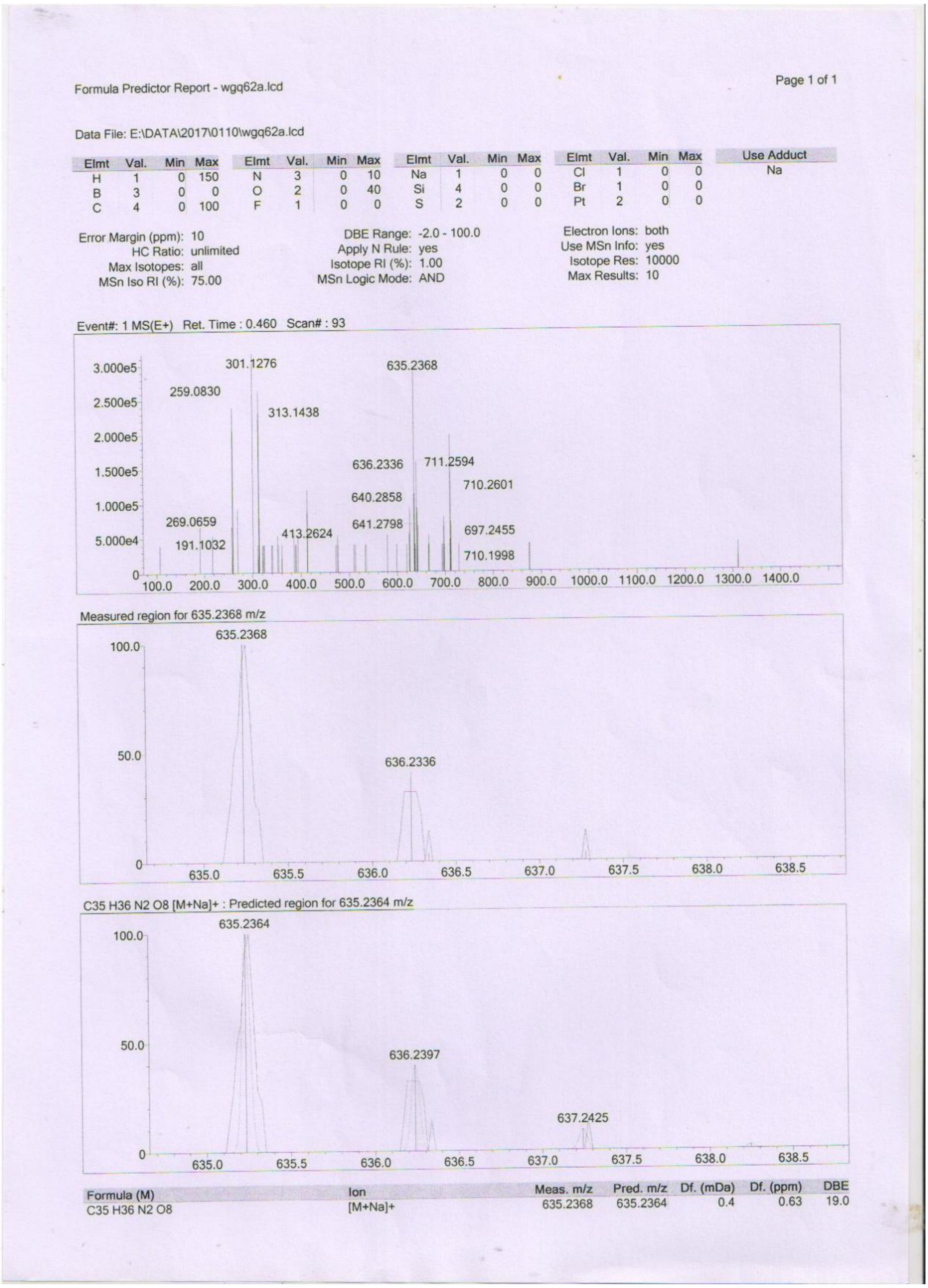

Formula Predictor Report - wgq62a.lcd

Page 1 of 1

Data File: E:\DATA\2017\0110\wgq62a.lcd

Elmt	Val.	Min	Max	Elmt	Val.	Min	Max	Elmt	Val.	Min	Max	Elmt	Val.	Min	Max	Use Adduct
H	1	0	150	N	3	0	10	Na	1	0	0	Cl	1	0	0	Na
B	3	0	0	O	2	0	40	Si	4	0	0	Br	1	0	0	
C	4	0	100	F	1	0	0	S	2	0	0	Pt	2	0	0	

Error Margin (ppm): 10
HC Ratio: unlimited
Max Isotopes: all
MSn Iso RI (%): 75.00
DBE Range: -2.0 - 100.0
Apply N Rule: yes
Isotope RI (%): 1.00
MSn Logic Mode: AND
Electron Ions: both
Use MSn Info: yes
Isotope Res: 10000
Max Results: 10

Formula (M)	Ion	Meas. m/z	Pred. m/z	Df. (mDa)	Df. (ppm)	DBE
C35 H36 N2 O8	[M+Na]+	635.2368	635.2364	0.4	0.63	19.0

Figure S22. HRESIMS spectrum of compound **2**

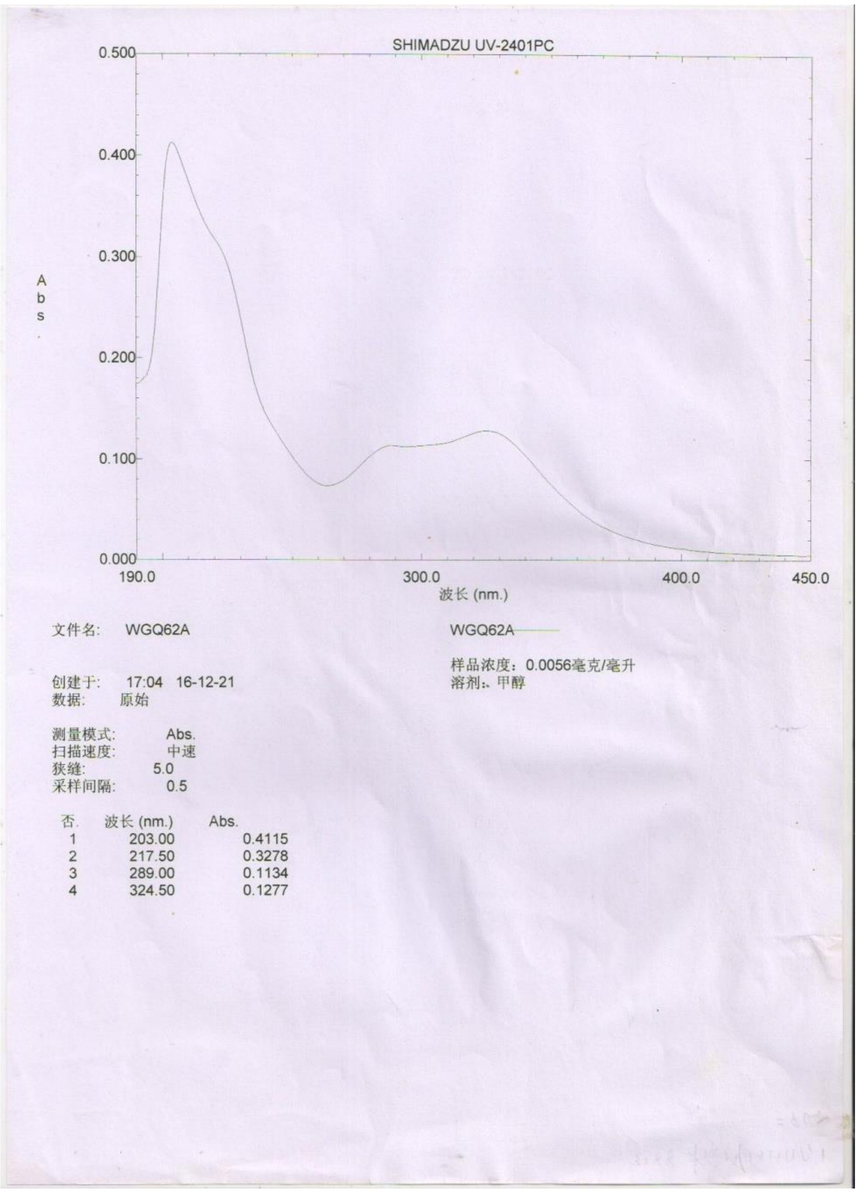

Figure S23. UV spectrum of compound **2**

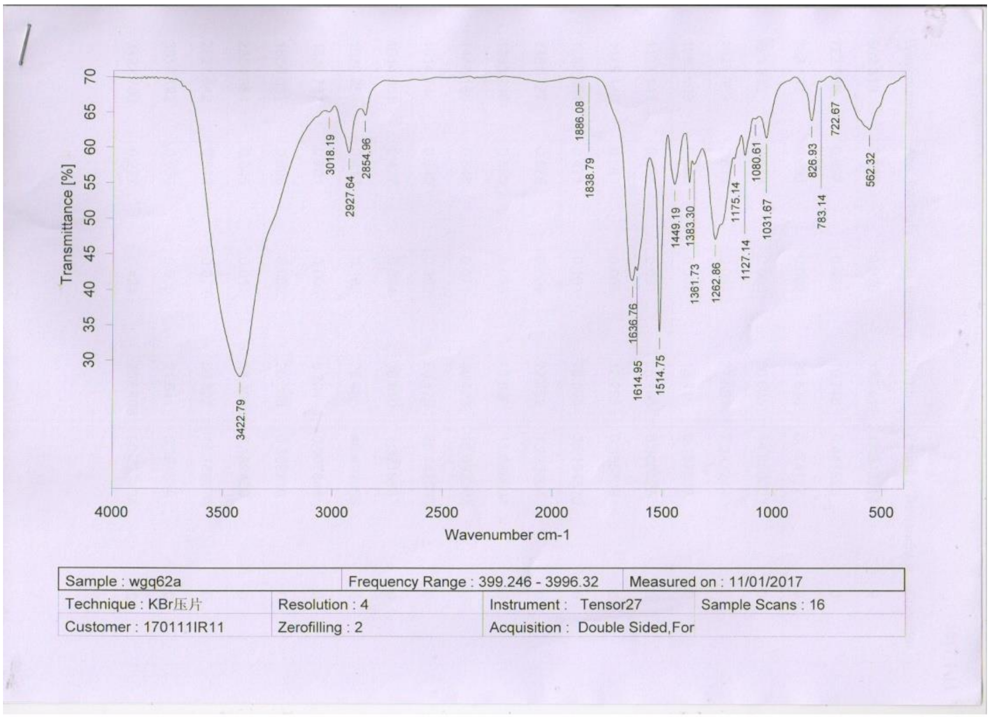

Figure S24. IR spectrum of compound **2**

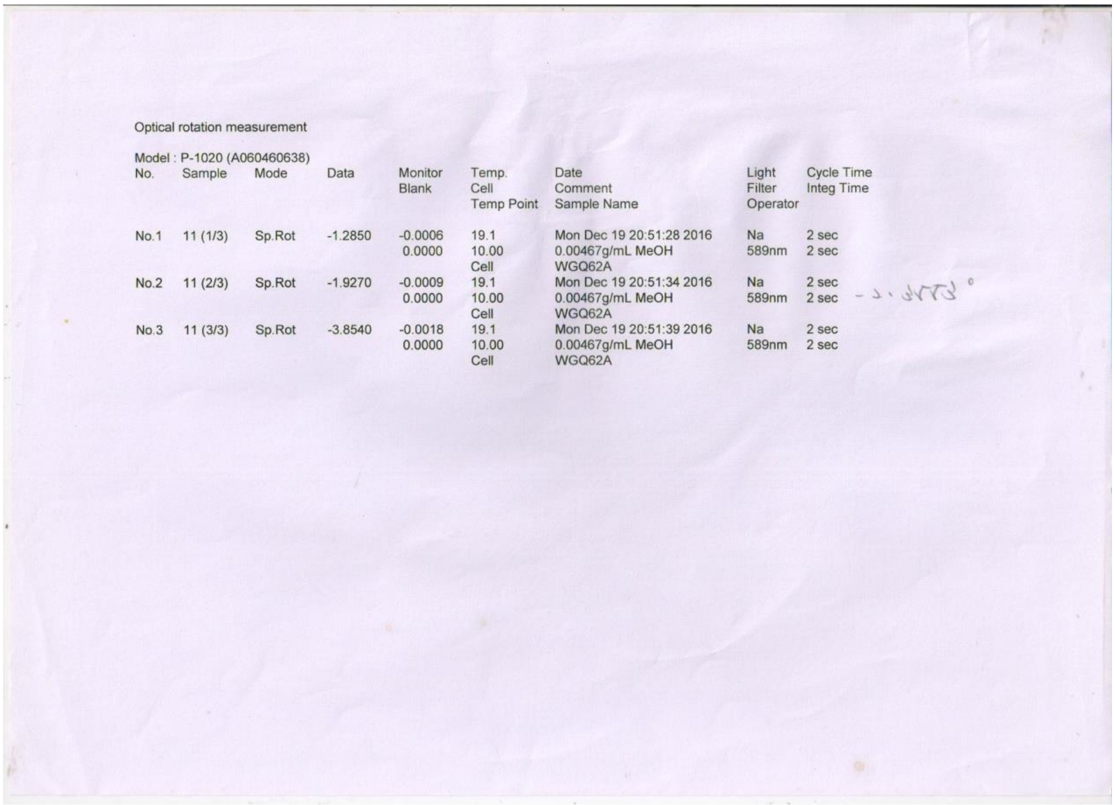

Optical rotation measurement

Model : P-1020 (A060460638)

No.	Sample	Mode	Data	Monitor<br>Blank	Temp.<br>Cell<br>Temp Point	Date<br>Comment<br>Sample Name	Light<br>Filter<br>Operator	Cycle Time<br>Integ Time
No.1	11 (1/3)	Sp.Rot	-1.2850	-0.0006<br>0.0000	19.1<br>10.00<br>Cell	Mon Dec 19 20:51:28 2016<br>0.00467g/mL MeOH<br>WGQ62A	Na<br>589nm	2 sec<br>2 sec
No.2	11 (2/3)	Sp.Rot	-1.9270	-0.0009<br>0.0000	19.1<br>10.00<br>Cell	Mon Dec 19 20:51:34 2016<br>0.00467g/mL MeOH<br>WGQ62A	Na<br>589nm	2 sec<br>2 sec
No.3	11 (3/3)	Sp.Rot	-3.8540	-0.0018<br>0.0000	19.1<br>10.00<br>Cell	Mon Dec 19 20:51:39 2016<br>0.00467g/mL MeOH<br>WGQ62A	Na<br>589nm	2 sec<br>2 sec

Figure S25. Optical rotation of compound **2**

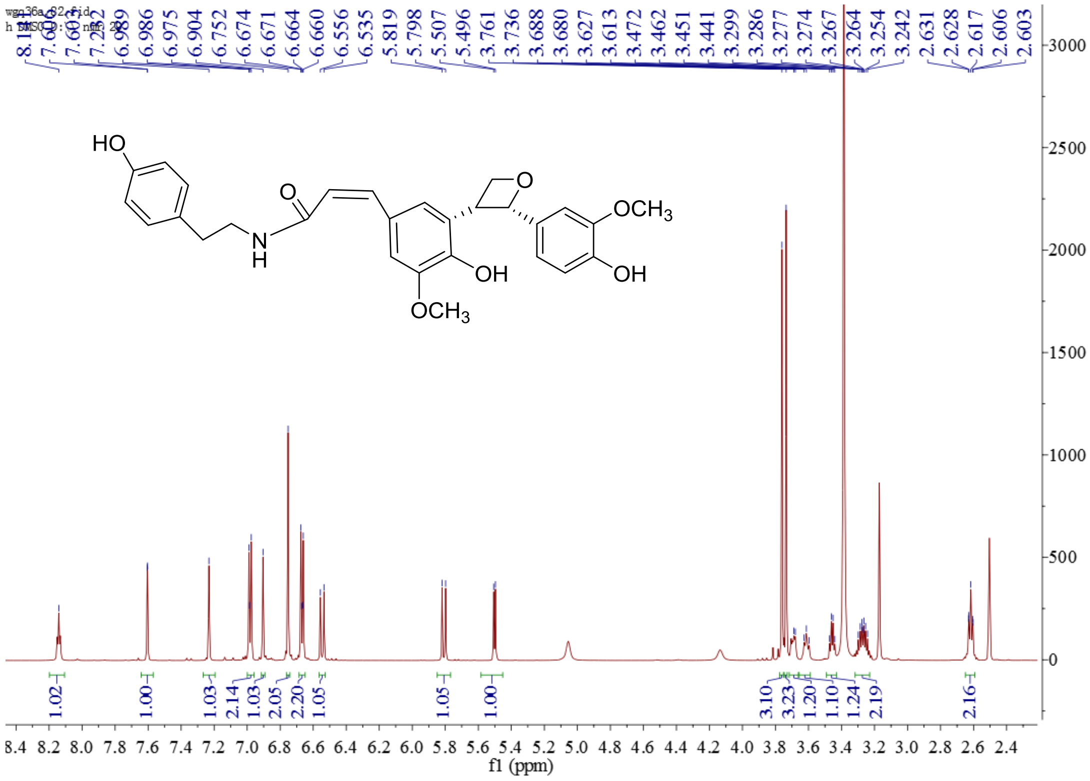

Figure S26. ^{1}H NMR spectrum of compound **3**

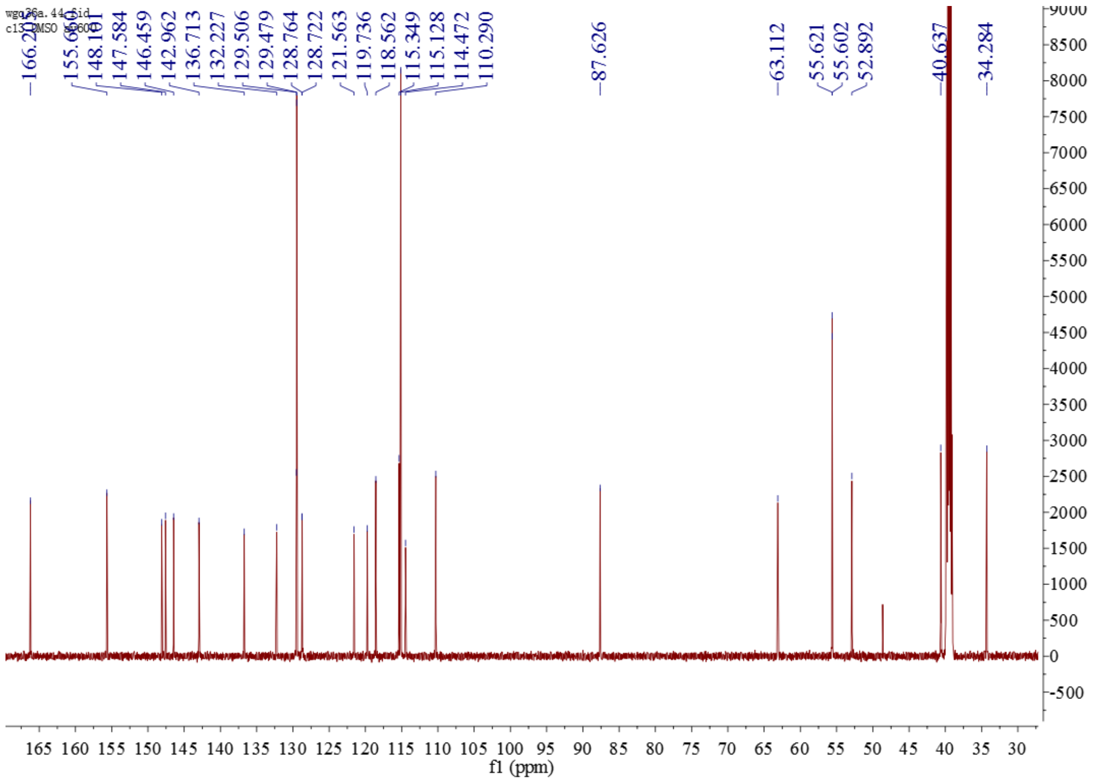

Figure S27. ^{13}C NMR spectrum of compound **3**

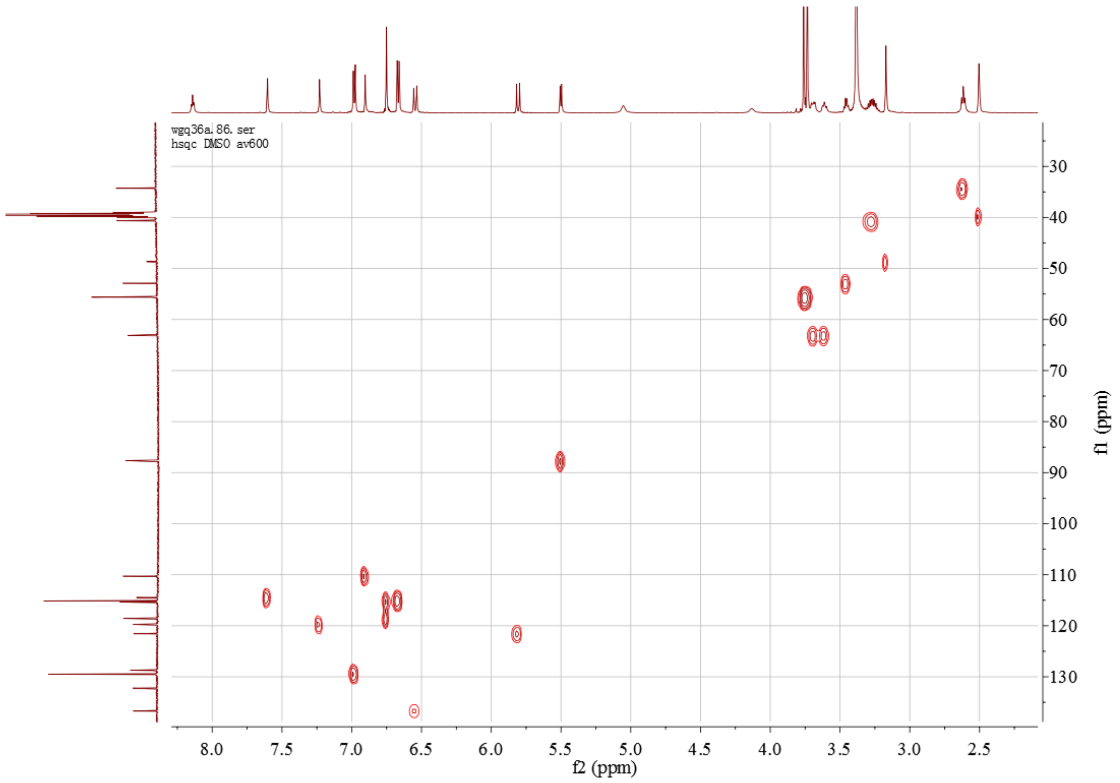

Figure S28. HSQC spectrum of compound **3**

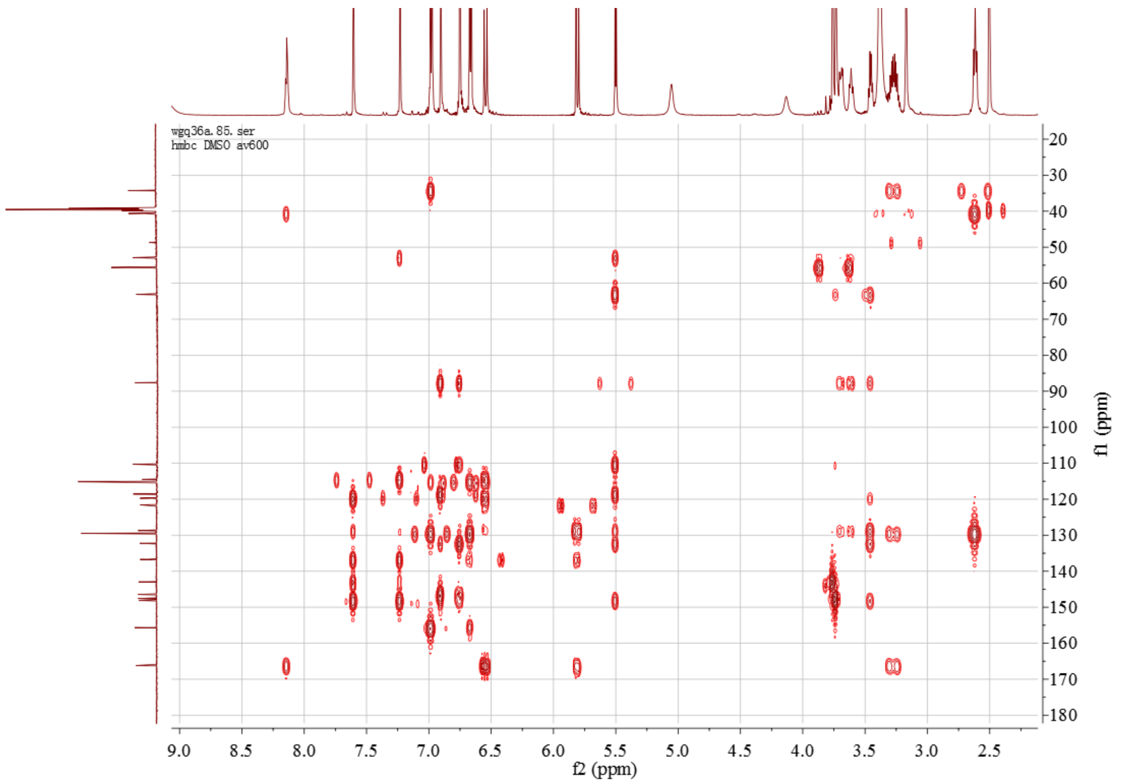

Figure S29. HMBC spectrum of compound **3**

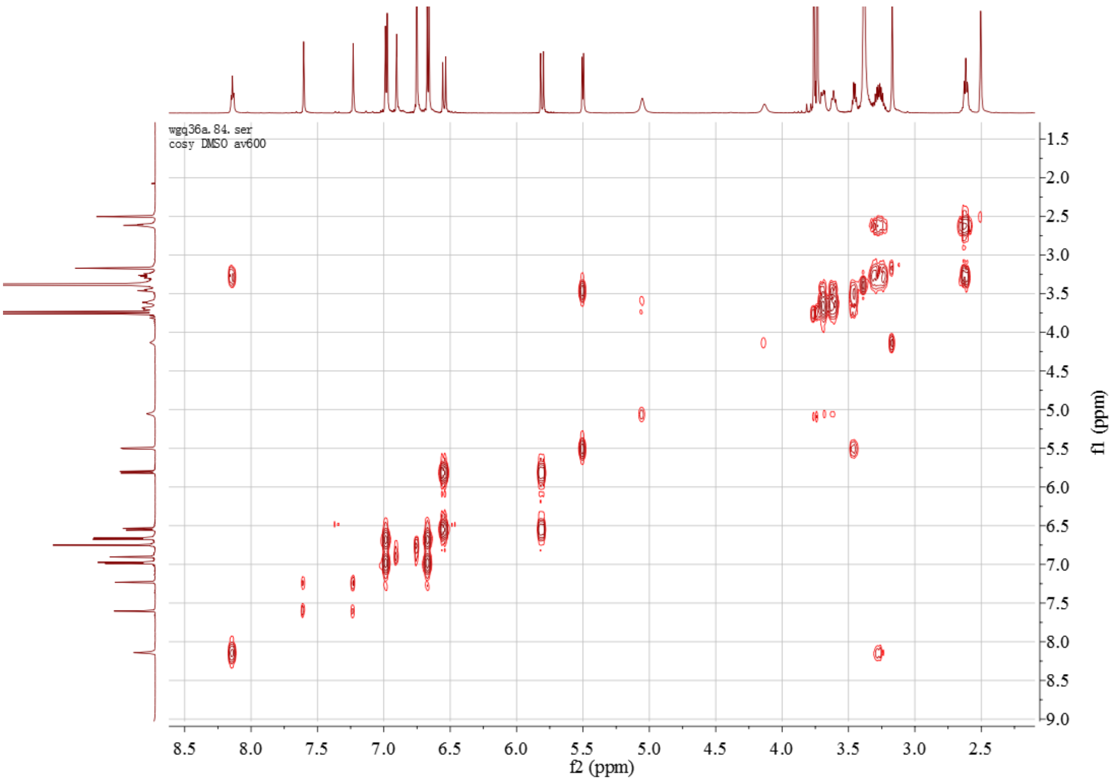

Figure S30. COSY spectrum of compound **3**

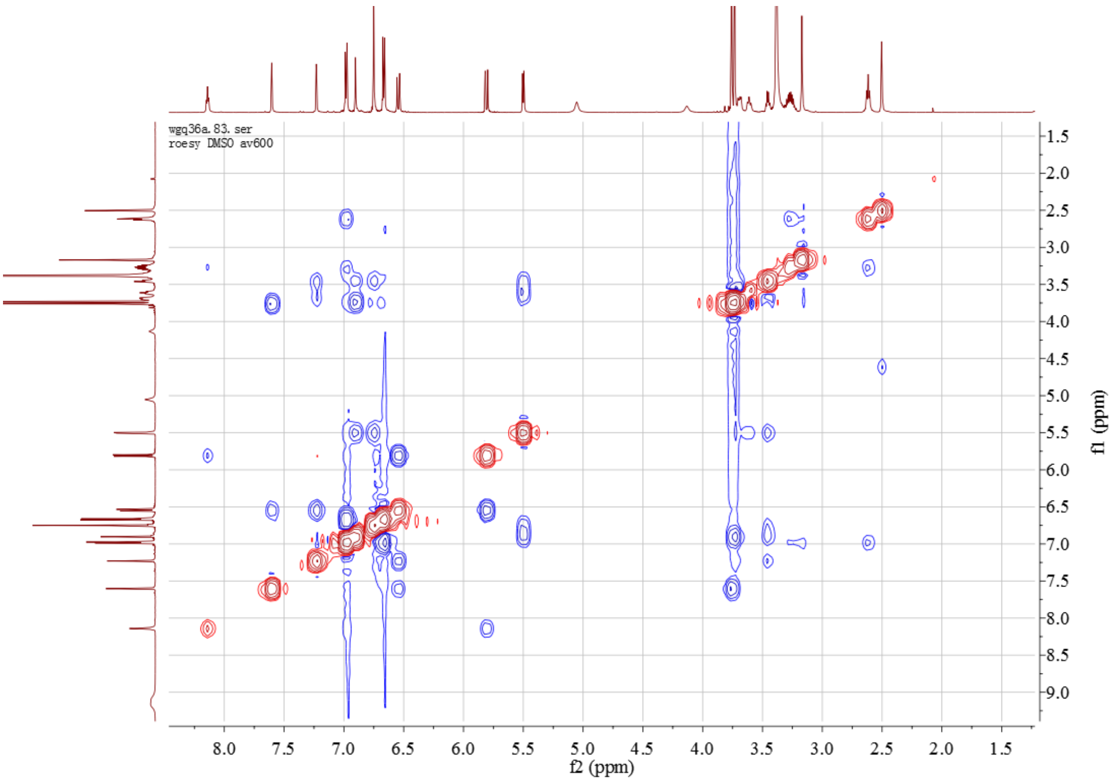

Figure S31. ROESY spectrum of compound**3**

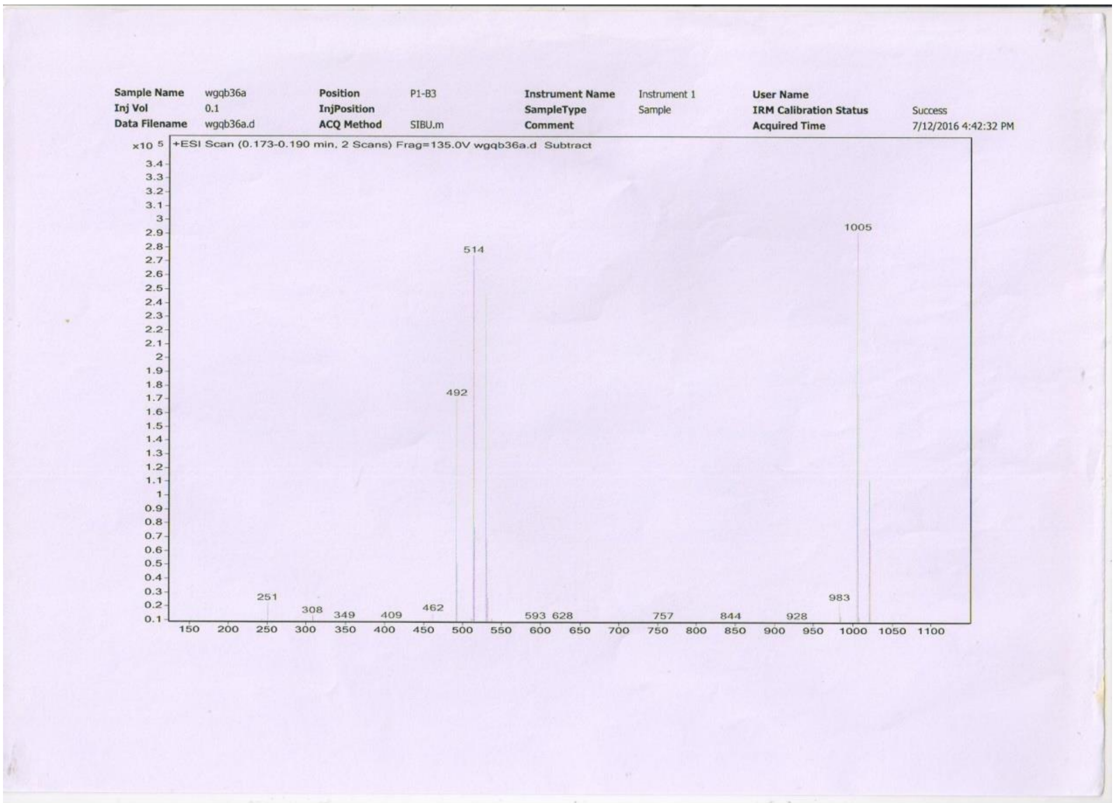

Figure S32. ESIMS spectrum of compound **3**

Qualitative Analysis Report

**Data Filename**	wgq36a.d	**Sample Name**	wgq36a
**Sample Type**	Sample	**Position**	P1-C4
**Instrument Name**	Instrument 1	**User Name**	
**Acq Method**	SIBU.m	**Acquired Time**	10/18/2016 3:52:12 PM
**IRM Calibration Status**	Success	**DA Method**	ESI+.m
**Comment**			

Sample Group **Info.**

Acquisition SW Version 6200 series TOF/6500 series Q-TOF B.05.01 (B5125.2)

User Spectra

Fragmentor Voltage	Collision Energy	Ionization Mode
175	0	ESI

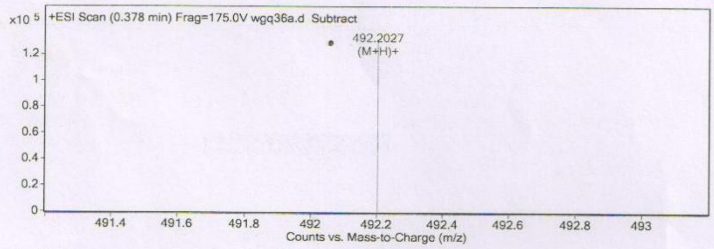

Peak List

m/z	z	Abund	Formula	Ion
250.5723	2	17466.32		
492.2027	1	131583.2	C28 H29 N O7	(M+H)+
493.206	1	38735.98	C28 H29 N O7	(M+H)+
514.1845	1	63947.07		
515.1874	1	19022.08		
530.1583	1	46037.93		
531.1604	1	13850		
1005.3815	1	12516.89		

Formula Calculator Element Limits

Element	Min	Max
C	3	60
H	0	120
O	0	30
N	0	5

Formula Calculator Results

Formula	CalculatedMass	CalculatedMz	Mz	Diff. (mDa)	Diff. (ppm)	DBE
C28 H29 N O7	491.1944	492.2017	492.2027	-1.0	-2.1	15.0000

--- End Of Report ---

Figure S33. HRESIMS spectrum of compound **3**

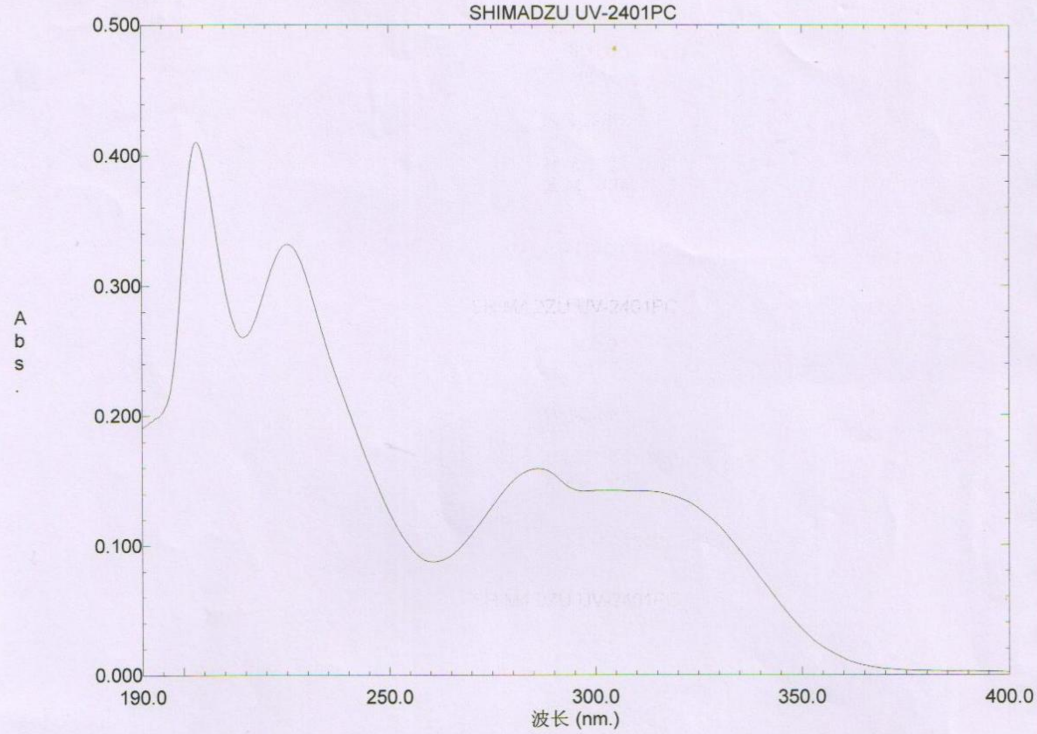

文件名:　WGQ36A

WGQ36A

样品浓度：0.0046毫克/毫升
溶剂：甲醇

创建于:　16:10　16-07-28
数据:　原始

测量模式:　Abs.
扫描速度:　中速
狭缝:　5.0
采样间隔:　0.2

否.	波长 (nm.)	Abs.
1	304.00	0.1419
2	286.20	0.1588
3	225.20	0.3316
4	203.40	0.4104

Figure S34. UV spectrum of compound **3**

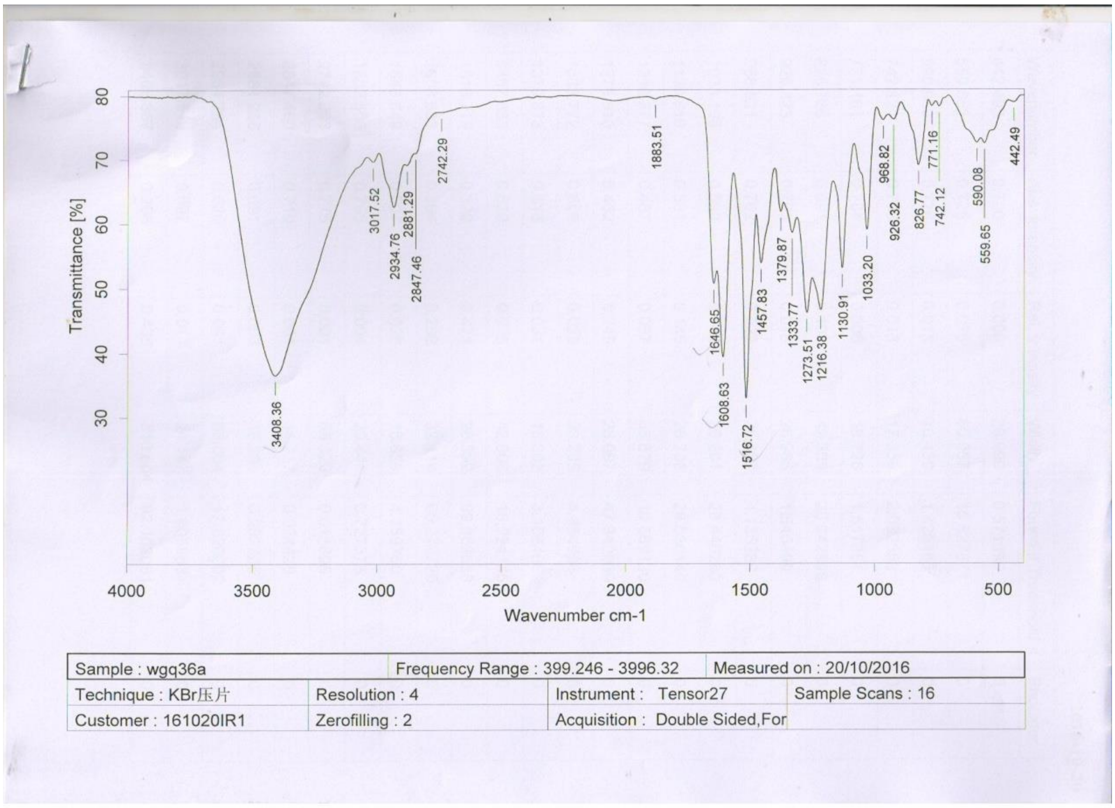

Figure S35. IR spectrum of compound **3**

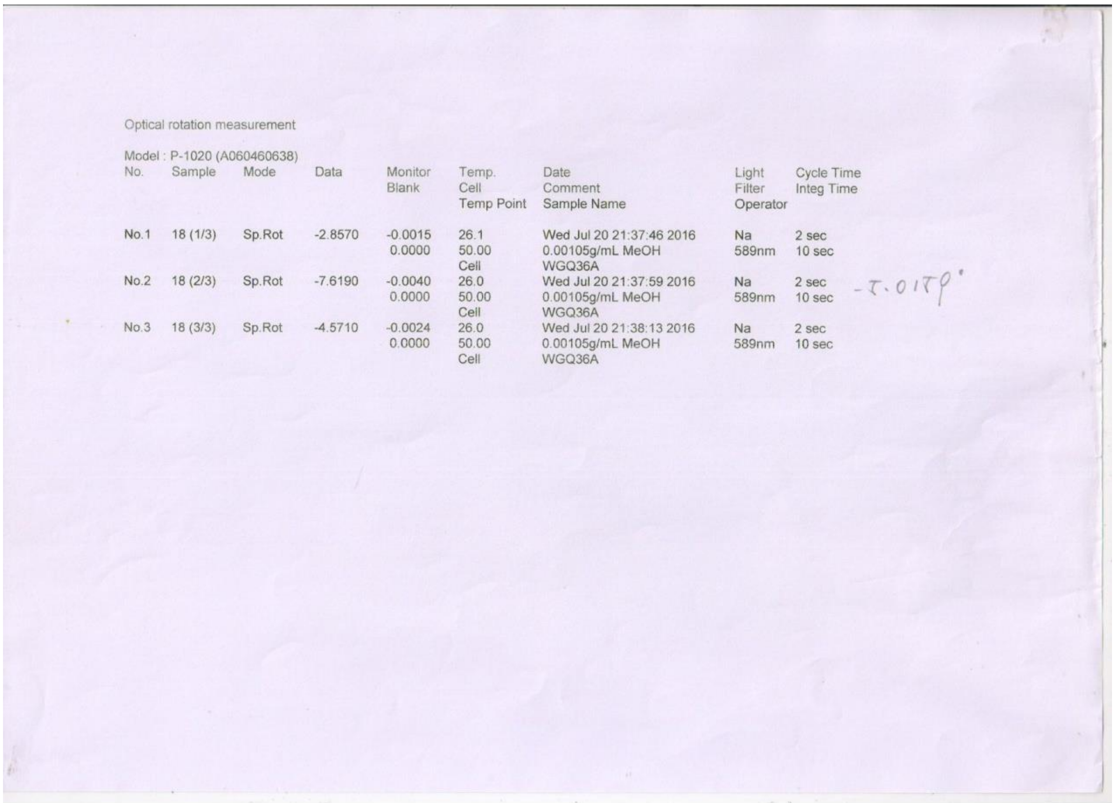

Optical rotation measurement

Model : P-1020 (A060460638)

No.	Sample	Mode	Data	Monitor<br>Blank	Temp.<br>Cell<br>Temp Point	Date<br>Comment<br>Sample Name	Light<br>Filter<br>Operator	Cycle Time<br>Integ Time
No.1	18 (1/3)	Sp.Rot	-2.8570	-0.0015<br>0.0000	26.1<br>50.00<br>Cell	Wed Jul 20 21:37:46 2016<br>0.00105g/mL MeOH<br>WGQ36A	Na<br>589nm	2 sec<br>10 sec
No.2	18 (2/3)	Sp.Rot	-7.6190	-0.0040<br>0.0000	26.0<br>50.00<br>Cell	Wed Jul 20 21:37:59 2016<br>0.00105g/mL MeOH<br>WGQ36A	Na<br>589nm	2 sec<br>10 sec
No.3	18 (3/3)	Sp.Rot	-4.5710	-0.0024<br>0.0000	26.0<br>50.00<br>Cell	Wed Jul 20 21:38:13 2016<br>0.00105g/mL MeOH<br>WGQ36A	Na<br>589nm	2 sec<br>10 sec

-5.0159°

Figure S36. Optical rotation of compound **3**

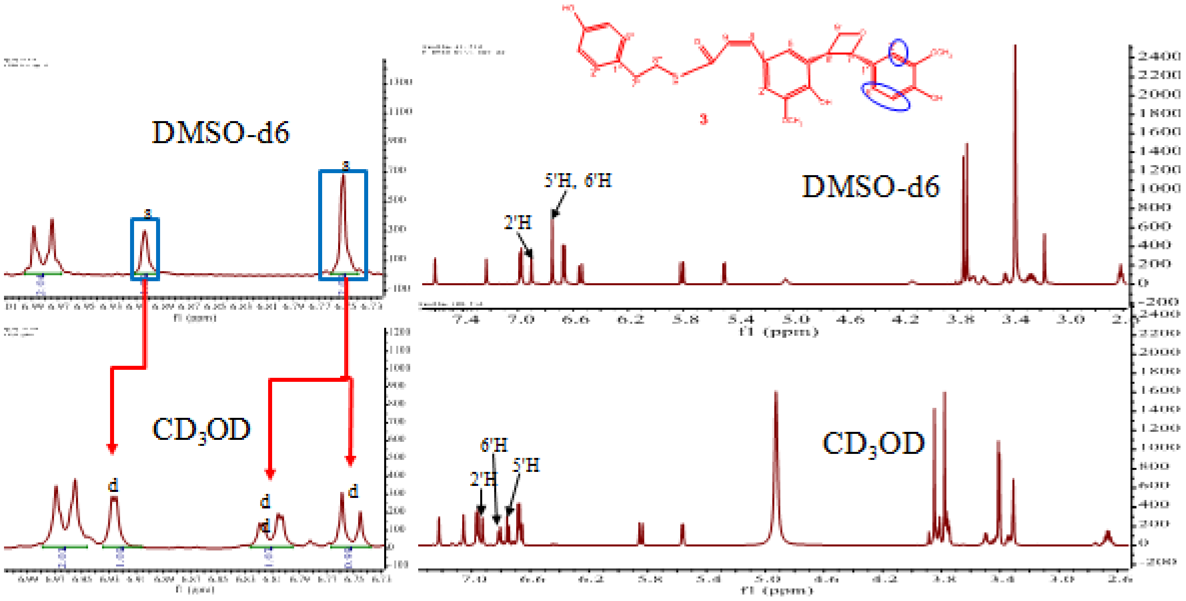

Figure S37. Influence of deuterated solvent on the ^{1}H NMR spectra of compound **3**

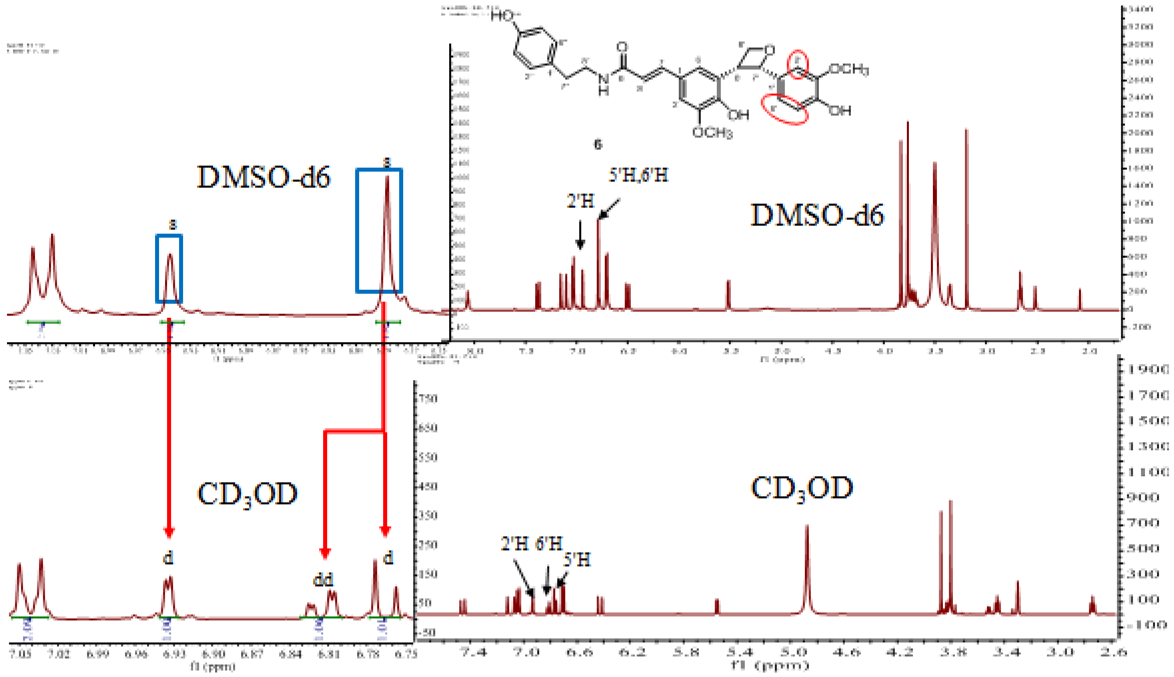

Figure S38. Influence of deuterated solvent on the ^{1}H NMR spectra of compound **6**

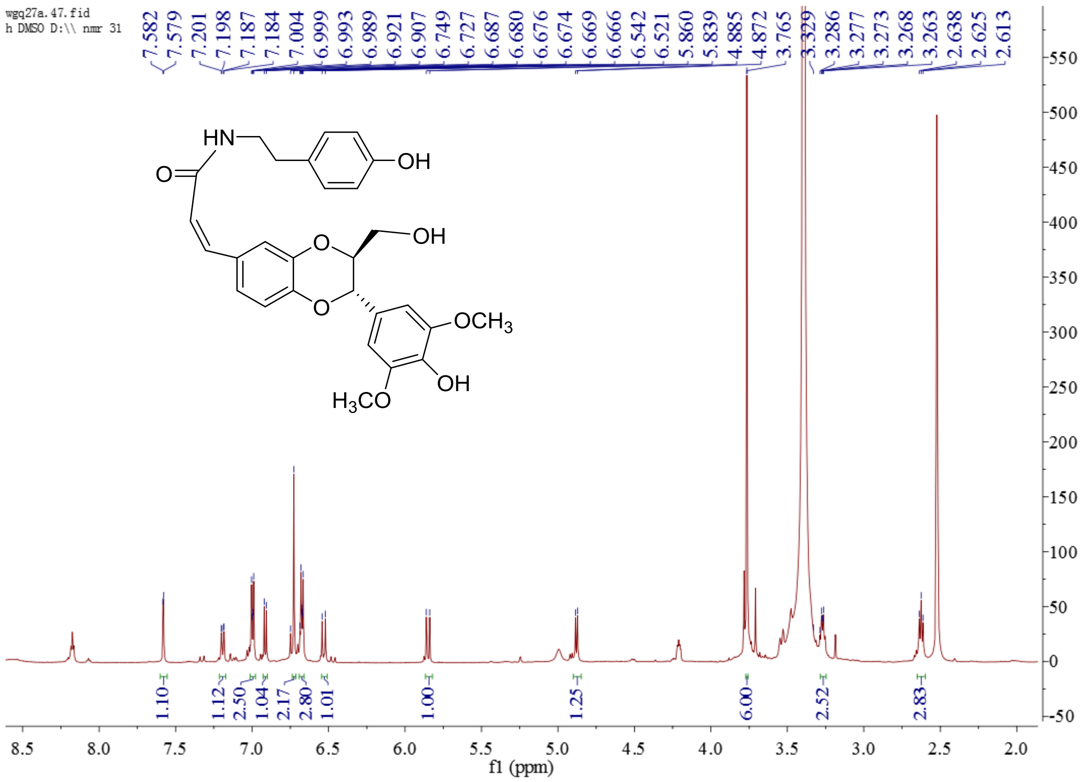

Figure S39.^{1}H NMR spectrum of compound **4**

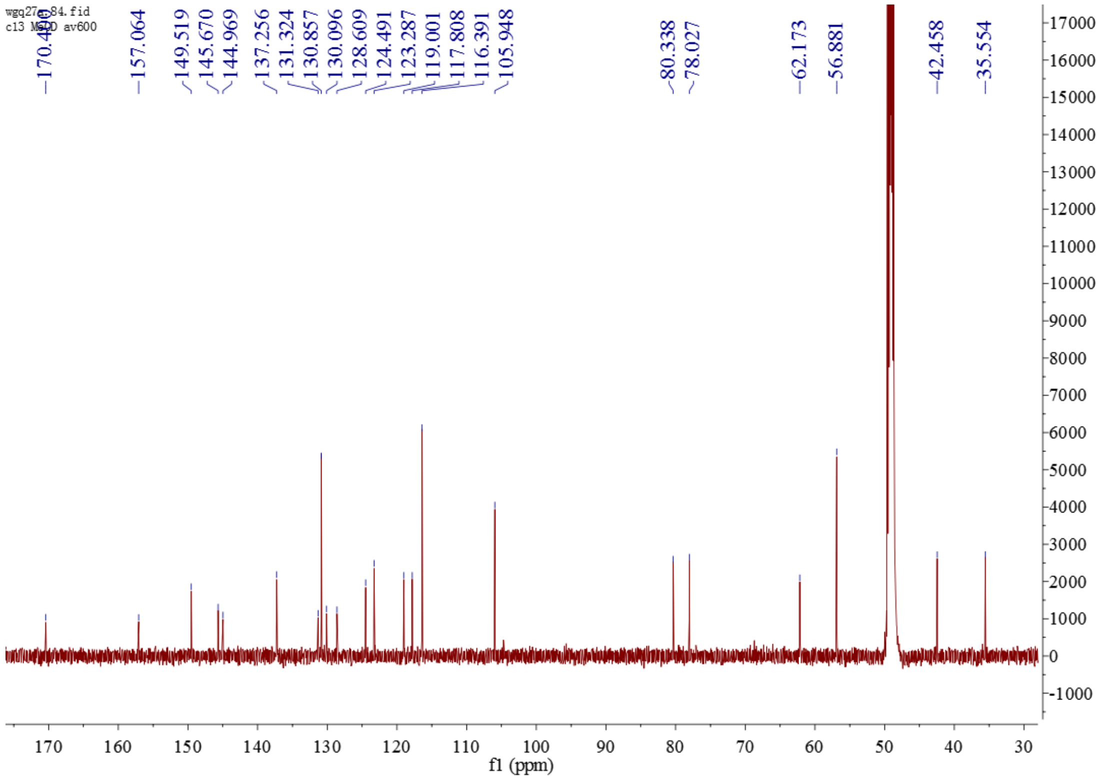

Figure S40.^{13}C NMR spectrum of compound **4**

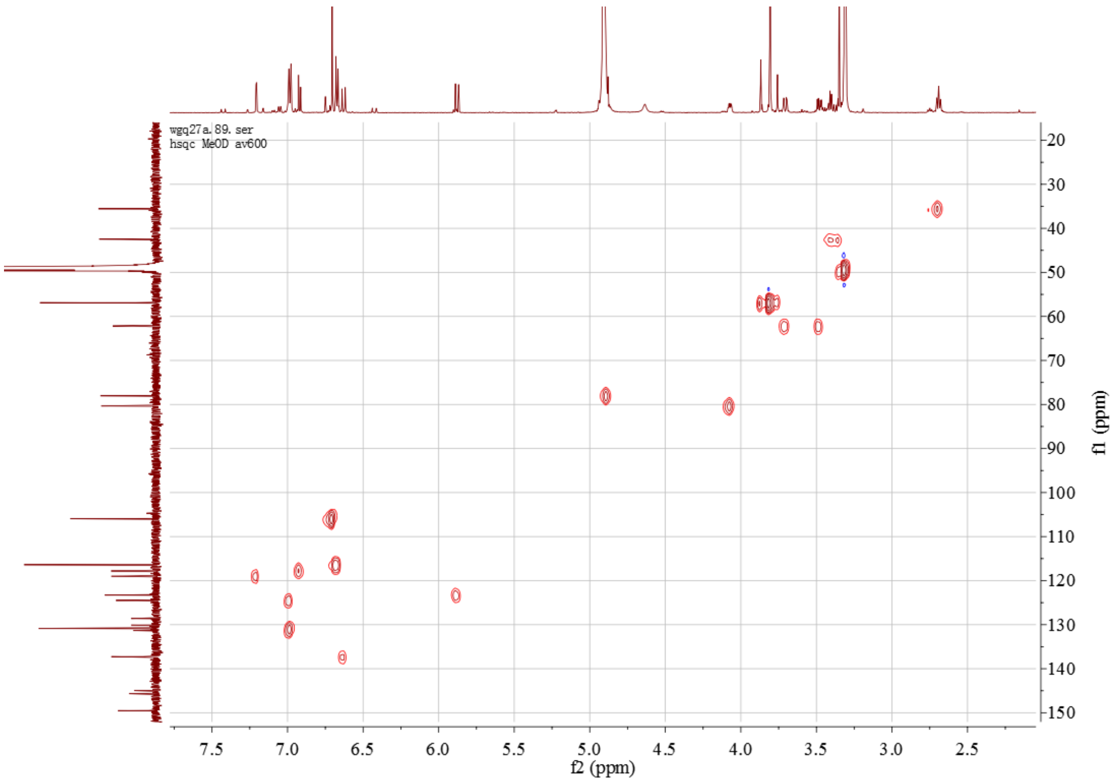

Figure S41. HSQC spectrum of compound **4**

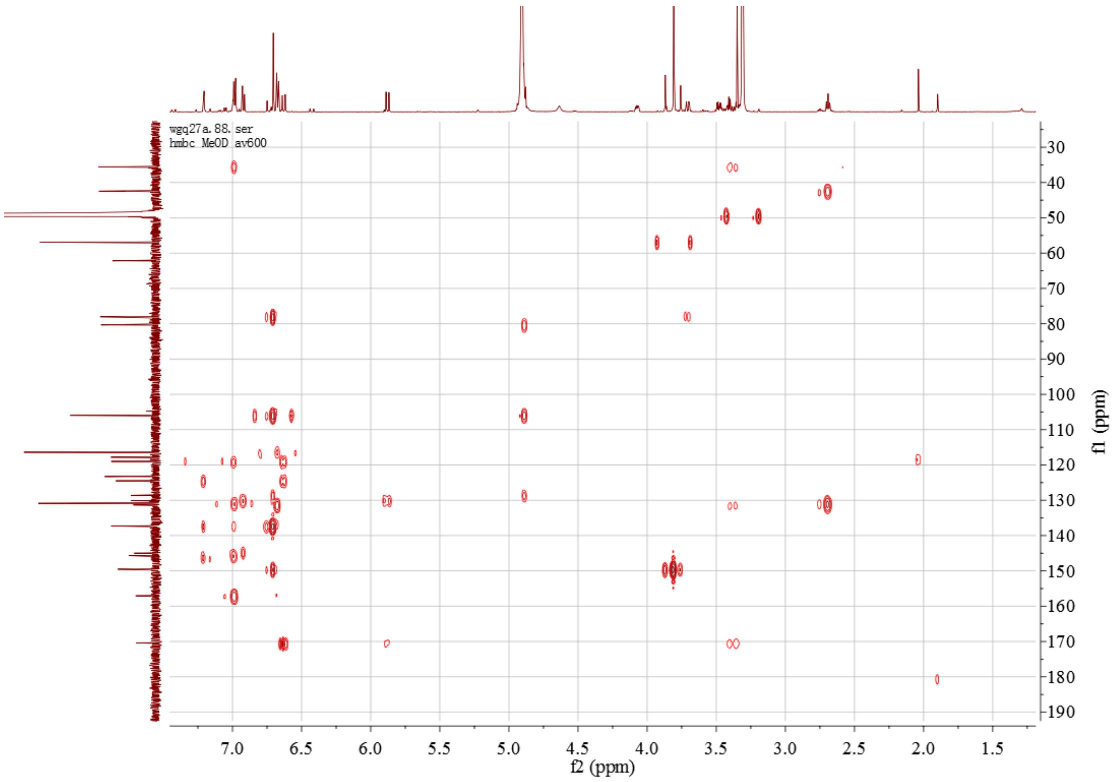

Figure S42. HMBC spectrum of compound **4**

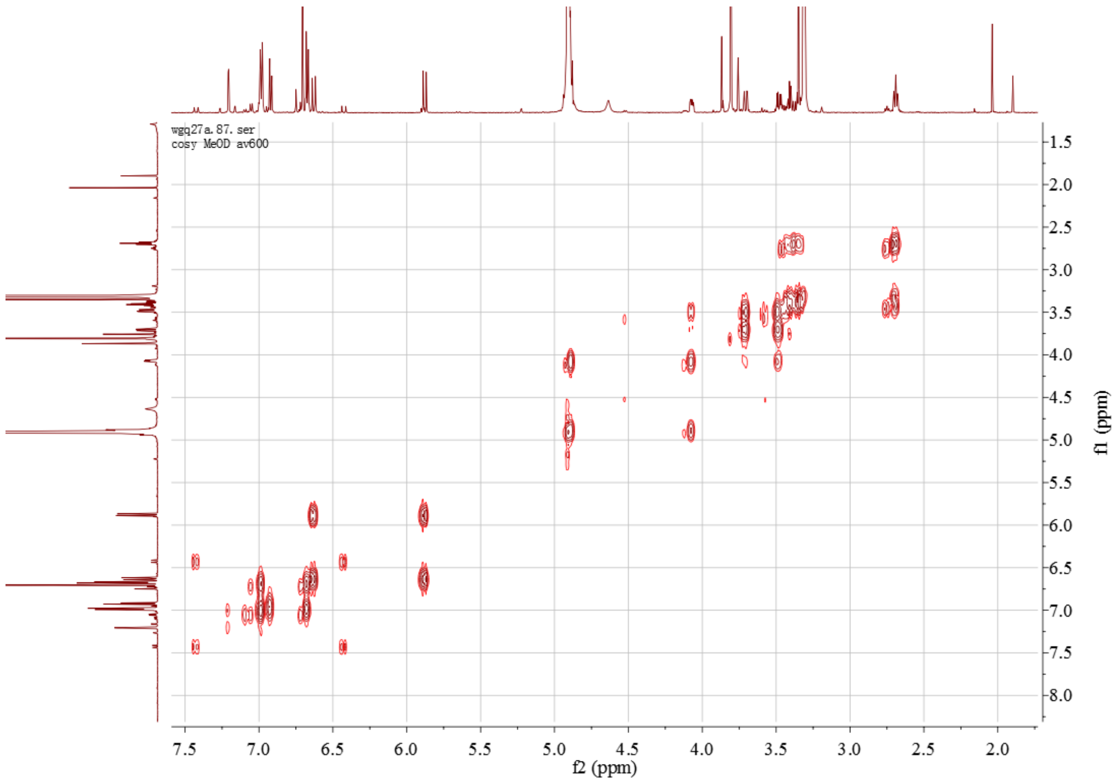

Figure S43.COSY spectrum of compound **4**

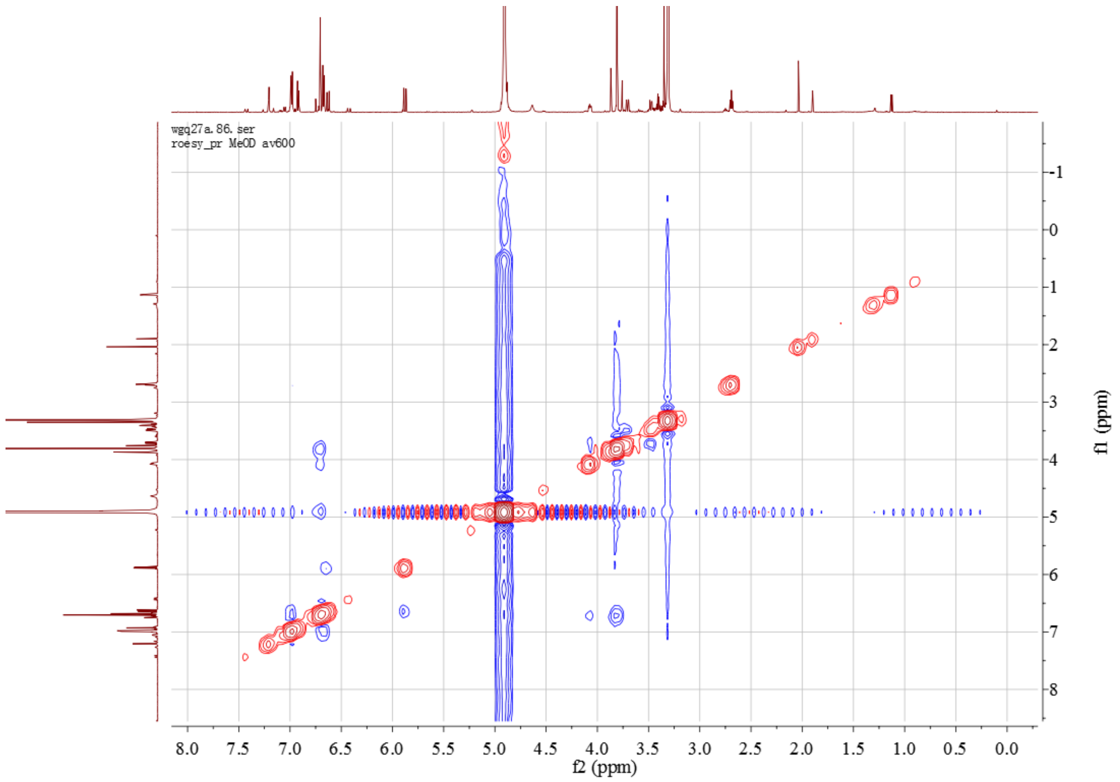

Figure S44.ROESY spectrum of compound **4**

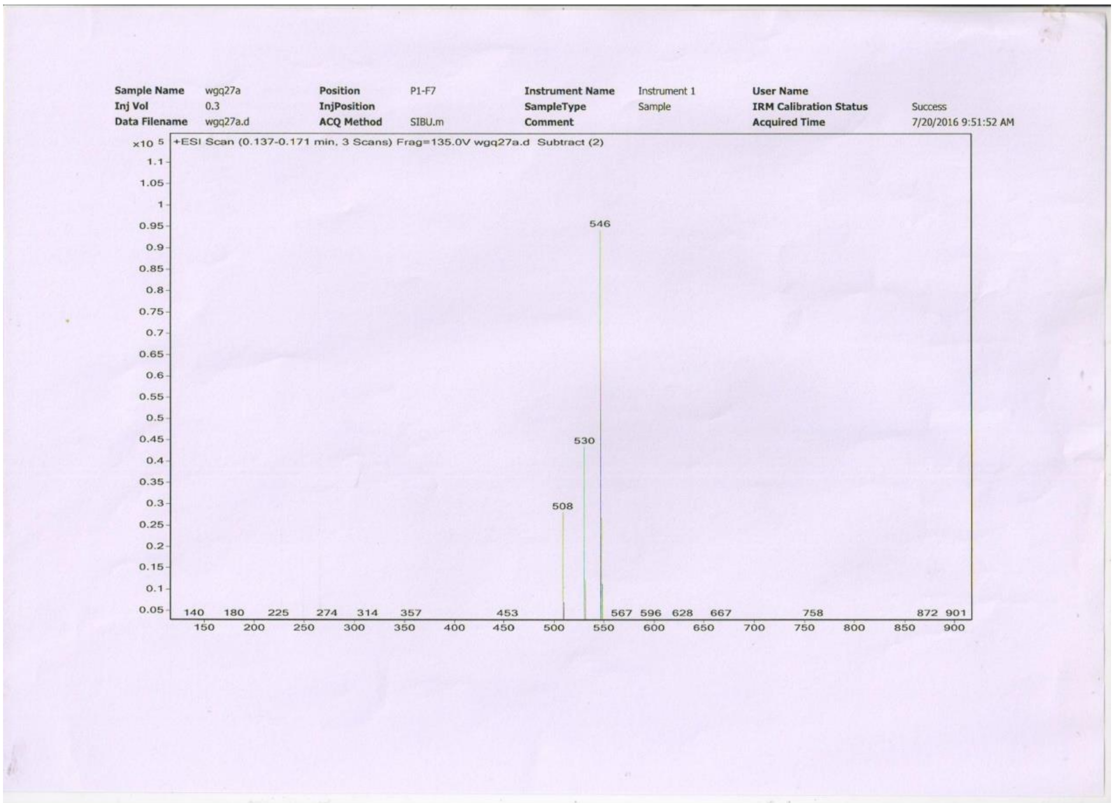

Figure S45. ESIMS spectrum of compound **4**

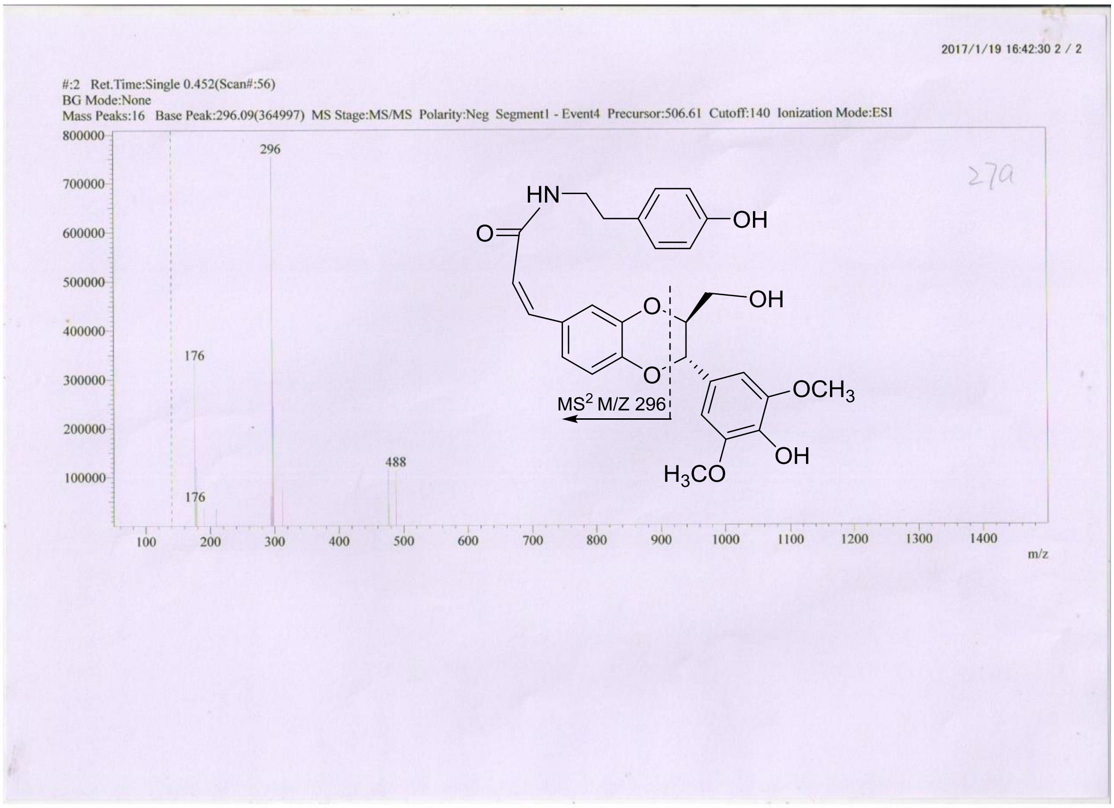

Figure S46. ESIMS/MS spectrum of compound **4**

Formula Predictor Report - wgq27a.lcd

Data File: E:\DATA\2017\0110\wgq27a.lcd

Elmt	Val.	Min	Max	Elmt	Val.	Min	Max	Elmt	Val.	Min	Max	Elmt	Val.	Min	Max	Use Adduct
H	1	0	150	N	3	0	20	Na	1	0	0	Cl	1	0	5	H
B	3	0	0	O	2	0	40	Si	4	0	0	Br	1	0	0	
C	4	0	100	F	1	0	0	S	2	0	0	Pt	2	0	0	

Error Margin (ppm): 10
HC Ratio: unlimited
Max Isotopes: all
MSn Iso RI (%): 75.00

DBE Range: -2.0 - 100.0
Apply N Rule: yes
Isotope RI (%): 1.00
MSn Logic Mode: AND

Electron Ions: both
Use MSn Info: yes
Isotope Res: 10000
Max Results: 10

Event#: 2 MS(E-) Ret. Time : 0.350 Scan# : 72

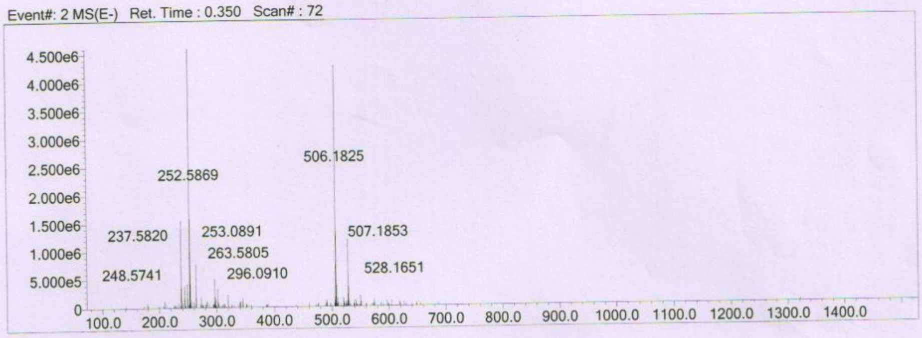

Measured region for 506.1825 m/z

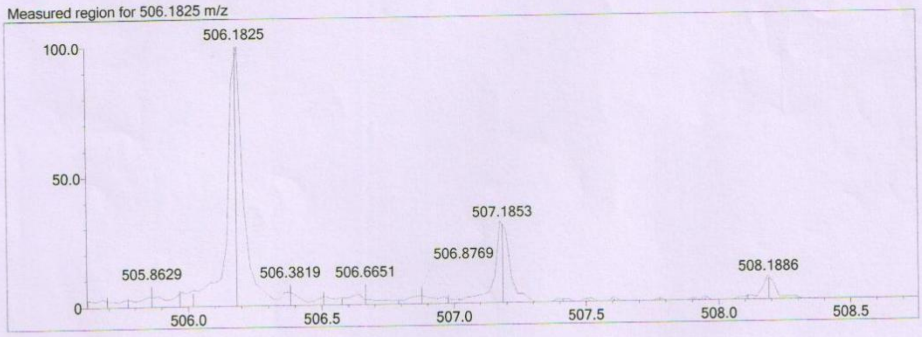

C28 H29 N O8 [M-H]- : Predicted region for 506.1820 m/z

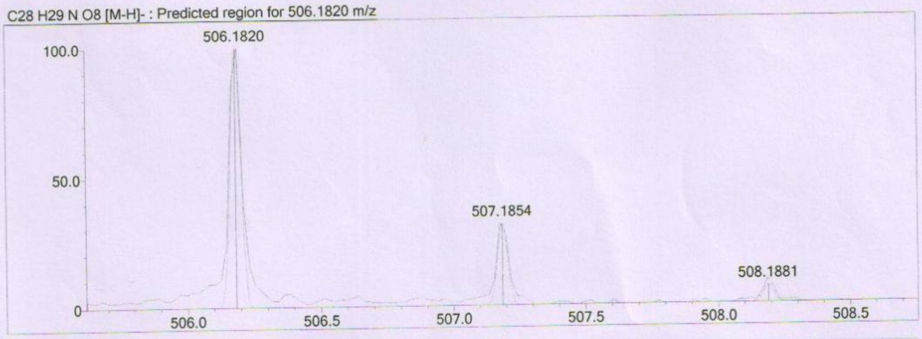

Formula (M)	Ion	Meas. m/z	Pred. m/z	Df. (mDa)	Df. (ppm)	DBE
C28 H29 N O8	[M-H]-	506.1825	506.1820	0.5	0.99	15.0

Figure S47. HRESIMS spectrum of compound **4**

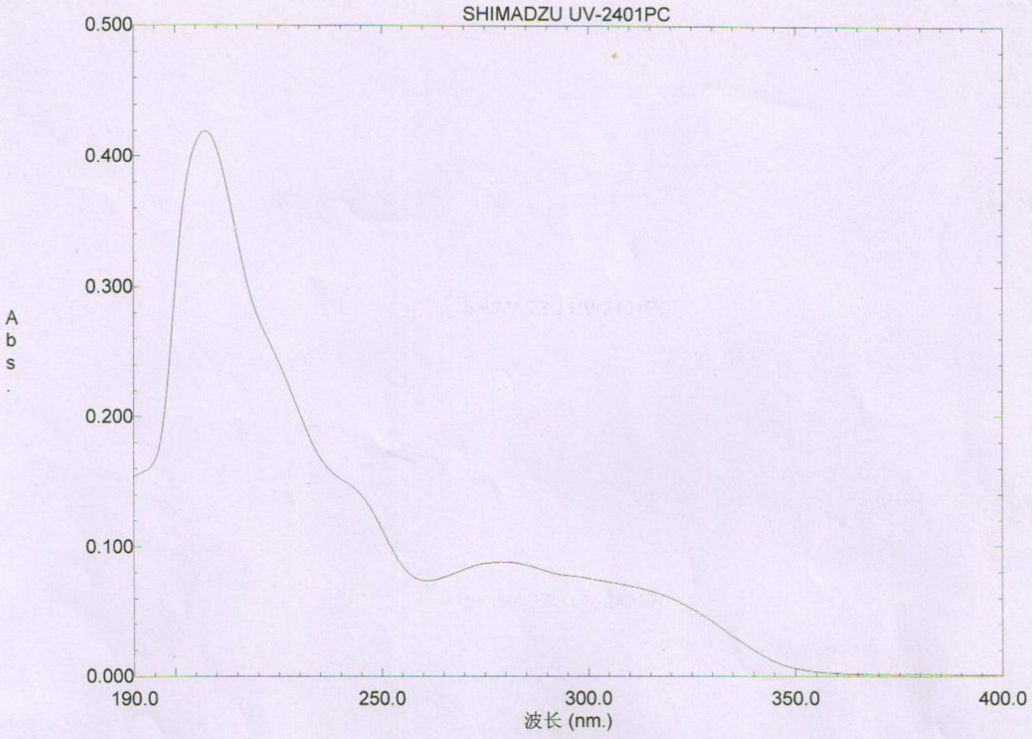

文件名: WGQ27A

WGQ27A

样品浓度：0.0043毫克/毫升
溶剂：甲醇

创建于: 16:31 16-11-14
数据: 原始

测量模式: Abs.
扫描速度: 中速
狭缝: 5.0
采样间隔: 0.5

否.	波长 (nm.)	Abs.
1	207.00	0.4191
2	240.00	0.1520
3	278.50	0.0878

Figure S48. UV spectrum of compound **4**

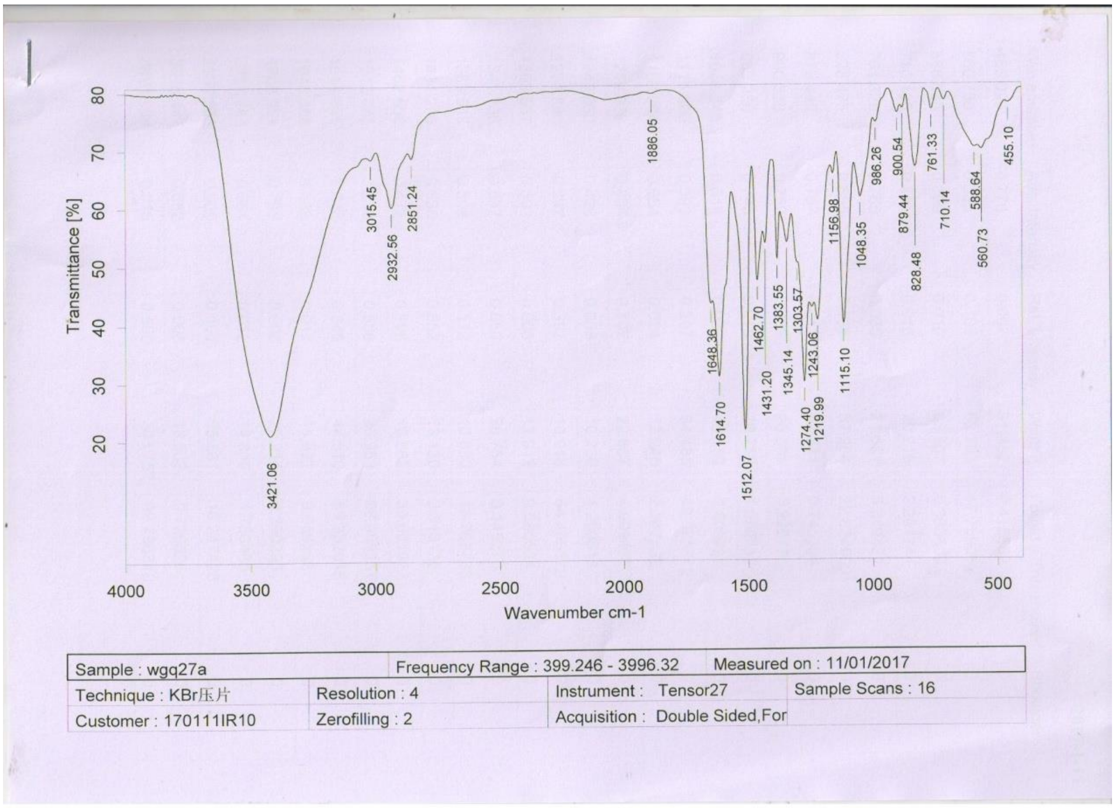

Figure S49. IR spectrum of compound **4**

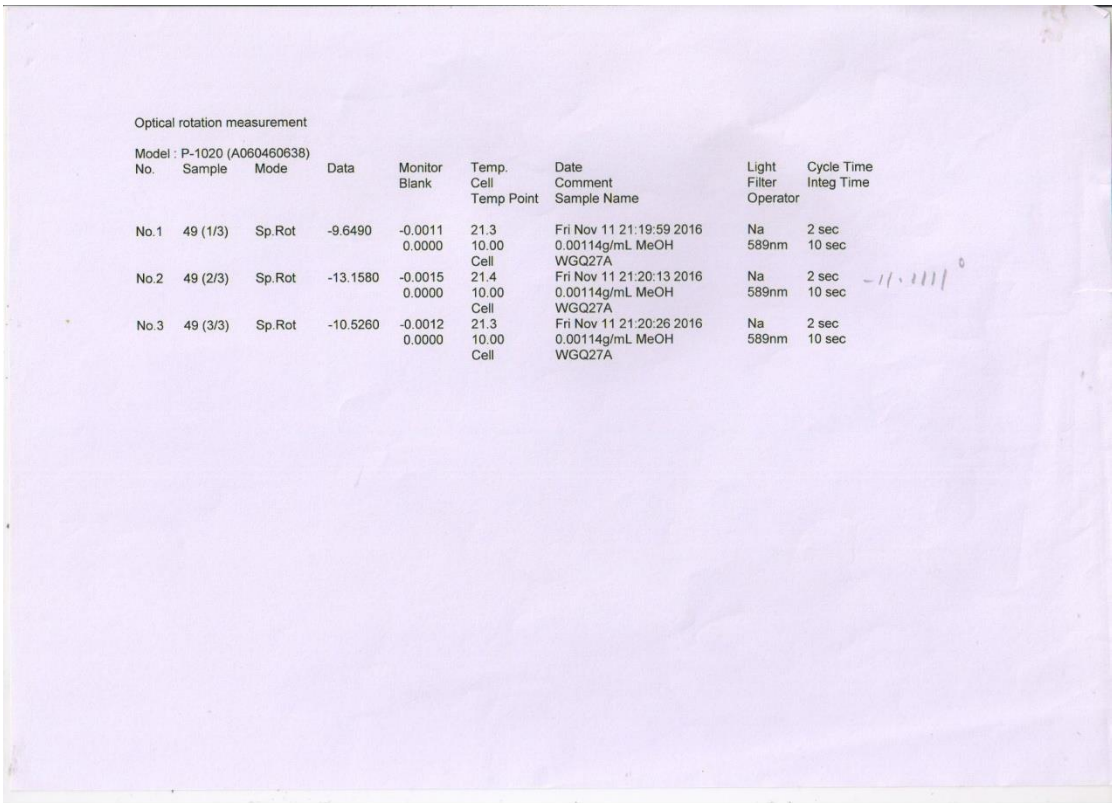

Optical rotation measurement

Model : P-1020 (A060460638)

No.	Sample	Mode	Data	Monitor<br>Blank	Temp.<br>Cell<br>Temp Point	Date<br>Comment<br>Sample Name	Light<br>Filter<br>Operator	Cycle Time<br>Integ Time
No.1	49 (1/3)	Sp.Rot	-9.6490	-0.0011<br>0.0000	21.3<br>10.00<br>Cell	Fri Nov 11 21:19:59 2016<br>0.00114g/mL MeOH<br>WGQ27A	Na<br>589nm	2 sec<br>10 sec
No.2	49 (2/3)	Sp.Rot	-13.1580	-0.0015<br>0.0000	21.4<br>10.00<br>Cell	Fri Nov 11 21:20:13 2016<br>0.00114g/mL MeOH<br>WGQ27A	Na<br>589nm	2 sec<br>10 sec
No.3	49 (3/3)	Sp.Rot	-10.5260	-0.0012<br>0.0000	21.3<br>10.00<br>Cell	Fri Nov 11 21:20:26 2016<br>0.00114g/mL MeOH<br>WGQ27A	Na<br>589nm	2 sec<br>10 sec

-11.1111°

Figure S50. Optical rotation of compound **4**
